# Determination of in vivo RNA kinetics using RATE-seq

**DOI:** 10.1261/rna.045104.114

**Published:** 2014-10

**Authors:** Benjamin Neymotin, Rodoniki Athanasiadou, David Gresham

**Affiliations:** Center for Genomics and Systems Biology, Department of Biology, New York University, New York, New York 10003, USA

**Keywords:** RATE-seq, RNA degradation, RNA synthesis, thiouracil, metabolic labeling

## Abstract

The abundance of a transcript is determined by its rate of synthesis and its rate of degradation; however, global methods for quantifying RNA abundance cannot distinguish variation in these two processes. Here, we introduce RNA approach to equilibrium sequencing (RATE-seq), which uses in vivo metabolic labeling of RNA and approach to equilibrium kinetics, to determine absolute RNA degradation and synthesis rates. RATE-seq does not disturb cellular physiology, uses straightforward normalization with exogenous spike-ins, and can be readily adapted for studies in most organisms. We demonstrate the use of RATE-seq to estimate genome-wide kinetic parameters for coding and noncoding transcripts in *Saccharomyces cerevisiae*.

## INTRODUCTION

Remodeling of gene expression is critical for a broad range of biological processes from the cell division cycle and embryo development (Schier 2007) to cellular responses to extracellular signals (Gasch et al. 2000). Regulation of transcript abundance is controlled by the combined action of transcript synthesis and transcript degradation. Although the regulation of transcript synthesis has historically been the primary focus of investigation, there is accumulating evidence that RNA degradation plays an important role in dynamic biological processes (Elkon et al. 2010). A comprehensive understanding of the regulation of gene expression programs, and the development of mathematical models that explain the dynamics of gene expression, requires the accurate estimation of absolute rates of both RNA synthesis and RNA degradation in vivo.

A variety of high-throughput methods have been introduced with the goal of estimating in vivo rates of either RNA synthesis or degradation. Genomic run on assays (García-Martínez et al. 2004) provide a means of estimating mRNA synthesis rates; however, these methods require isolation of nuclei or permeabilization of cells, which likely compromises the physiology of cells. Until recently, mRNA decay rates have been estimated using transcriptional inhibition (Wang et al. 2002; Grigull et al. 2004; Shalem et al. 2008) using either temperature-sensitive alleles of RNA polymerase II or chemical inhibition of transcript production. While these methods succeed in inhibiting transcript synthesis, they typically result in a stress response or cellular death (Nonet et al. 1987) resulting in the estimation of mRNA decay rates that may have little physiological relevance.

Recently, methods using in vivo metabolic labeling of mRNAs (Cleary et al. 2005; Dölken et al. 2008) have been introduced using either the nucleobase 4-thiouracil (4tU) or nucleoside 4-thiouridine (4sU), which introduce a reactive thiol group into RNAs. Following RNA purification, the presence of a thiol group in RNAs enables conjugation to N-[6-(Biotinamido)hexyl]-3′-(2′-pyridyldithio)-propionamide (biotin-HPDP) and subsequent fractionation using streptavidin-coated magnetic beads. Genome-wide estimation of in vivo kinetic parameters using metabolic labeling of RNA with 4tU has been reported using different experimental designs. Pulse-chase labeling with 4tU (Munchel et al. 2011) represents a promising approach to estimating mRNA degradation rates. However, internal recycling of labeled nucleotides (Puckett et al. 1975; Nikolov and Dabeva 1985) may result in an incomplete chase thereby confounding the estimation of mRNA degradation rates. Alternatively, comparative Dynamic Transcriptome Analysis (cDTA) (Sun et al. 2012) (an updated version of Dynamic Transcriptome Analysis [DTA]) (Miller et al. 2011) estimates rates of mRNA degradation by determining the ratio of labeled to total RNA using hybridization to a DNA microarray at a single time point following addition of 4tU. However, cDTA requires the manufacture of customized dual species DNA microarrays to normalize hybridization signals, and relies on a single time-point after labeling, which may not accurately capture kinetic parameters. Indeed, the use of different individual time points has a significant effect on the estimated degradation rates for at least a subset of transcripts (Dölken et al. 2008), which is likely the case for similar approaches using RNA-seq (e.g., Schwanhäusser et al. 2011).

Here, we report a general method for accurate measurement of absolute RNA kinetic parameters in vivo. We use approach to equilibrium labeling (Greenberg 1972), which minimizes exposure of cells to 4tU and is not affected by nucleotide recycling. We undertook a series of rigorous controls to optimize each step of the RATE-seq method. By using strand-specific sequencing (Parkhomchuk et al. 2009) in combination with ribosomal depletion, we measured rates of decay for a variety of different types of RNA, including noncoding RNA and snRNA. We developed a normalization method using multiple spike-in RNAs that also enables identification and correction for technical artifacts. To account for the nature of the data (i.e., overdispersed count data in which the variance is greater than the mean) in model fitting we used a weighted nonlinear regression to estimate parameters. We used RATE-seq to define the regulatory landscape of steady-state transcript levels, defined as a function of the underlying kinetic parameters genome-wide, and find that many transcripts in budding yeast have similar steady-state levels but differ greatly in their rates of production and degradation. RATE-seq can be readily implemented in any organism, making it a generally applicable method for characterizing the steady-state in vivo kinetics of RNA with unprecedented resolution.

## RESULTS

### Thiouracil labeling follows approach to equilibrium kinetics

**FIGURE 1.**
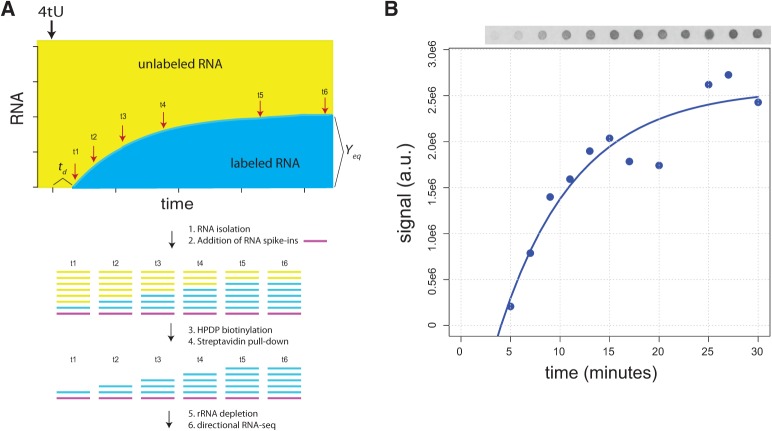
RATE-seq enables in vivo measurement of RNA kinetics. (*A*) Overview of approach to equilibrium labeling and analysis using RATE-seq. The increase in labeled transcript with time *Y*(*t*) modeled using the relationship 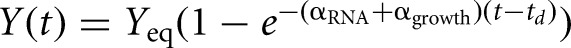, where *Y*_eq_ is the abundance of labeled transcript at steady state, α_RNA_ is the transcript's degradation rate constant, α_growth_ is the growth rate constant of the culture, *t* is the time after addition of label, and *t*_*d*_ is a time delay between the addition of label and the time at which labeled transcripts can be detected. Red arrows indicate points at which the RNA samples are recovered following addition of 4tU. (*B*) Incorporation of 4tU conforms to approach to equilbrium kinetics. An equivalent quantity of biotinylated polyadenylated RNA from timepoints following addition of 4tU was bound to a membrane and visualized using streptavidin alkaline phosphatase and chemifluorescence. Values are shown along with the model fit.

The rate of change in RNA abundance (*d*[RNA]/*dt*) can be modeled as a function of a constant rate of synthesis (*k*) and a degradation rate proportional to RNA abundance (α[RNA]) using the relationship *d*[RNA]/*dt* = *k*-α[RNA]. If a labeled nucleotide is added to the culture the concentration of labeled transcript will increase with time to an equilibrium value at a rate solely determined by the transcript's degradation rate constant (α_RNA_) and the cells’ division rate constant (α_growth_) (i.e., α = α_RNA_ + α_growth_). Approach to equilibrium labeling, using radiolabeling, was developed over 40 yr ago to estimate the rate of total mRNA turnover (Greenberg 1972) and was subsequently used to study individual transcripts using transcript-specific probes (Harpold et al. 1981; Kim and Warner 1983). To apply approach to equilibrium labeling on a genome-wide scale we developed a method using 4tU-labeling and RNA-seq (Fig. 1A). Our method relies on the presence of an endogenous copy of uracil phosphoribosyltransferase (UPRT) in *Saccharomyces cerevisiae* (encoded by *FUR1*), which converts 4tU into 4-thiouridine monophosphate allowing its incorporation in RNA. For the purpose of normalizing RNA-seq libraries from different time points following labeling, we added a constant quantity of three different in vitro-transcribed thiolated transcripts (Supplemental Table S1) to isolated RNA prior to fractionation. As deadenylated transcripts can persist in the cytoplasm or be readenylated in some species (Wilt 1973), we used rRNA depletion rather than poly(A) fractionation. The lack of poly(A) selection step also enables the analysis of both coding and noncoding transcripts.

We first tested the efficiency of 4tU incorporation in *S. cerevisiae* and its physiological consequences. Consistent with previous reports (Munchel et al. 2011) we find that yeast cells take up 4tU provided in the growth medium and incorporate it into RNA (Supplemental Fig. S1). However, we find that cells lacking a functional uridine monophosphate biosynthetic pathway (i.e., *ura3*^−^) cannot grow when supplemented with 4tU alone (Supplemental Fig. S2), suggesting that highly thiolated transcripts are not tolerated by the cell. As we found comparable 4tU incorporation in a *ura3*^−^ strain and prototrophic strain (Supplemental Fig. S1) we performed all subsequent experiments in a prototrophic strain. Over the timescale and concentrations of 4tU used for RATE-seq we detect no effect on cell growth (Supplemental Fig. S3), although prolonged exposure and higher concentrations appear to have slight effects (Supplemental Fig. S4). We confirmed that the concentration of 4tU used for RATE-seq does not affect global gene expression (Supplemental Fig. S5). Using a dot blot and colorimetric assay (Materials and Methods), we find that the pool of newly synthesized mRNA approaches equilibrium consistent with a model of constant synthesis and exponential degradation (Fig. 1B). Consistent with expectation, the equilibrium value of labeled RNA differs with different concentrations of 4tU, but the kinetics of the approach to equilibrium is unaffected (Supplemental Fig. S6). As with radiolabeling experiments in mammalian cells (Greenberg 1972), the mRNA fraction approaches equilibrium faster than total RNA (Supplemental Fig. S7), which reflects the relative stability of rRNA compared with mRNA.

### Measurement of RNA degradation rates transcriptome-wide

**FIGURE 2.**
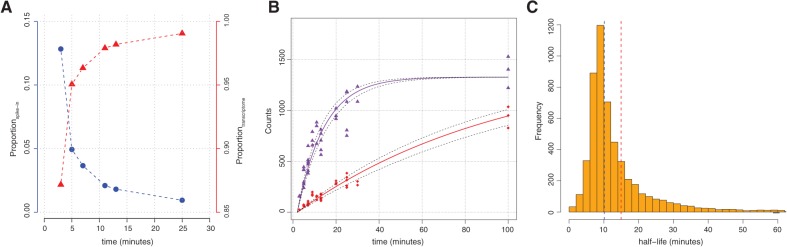
Global RNA kinetics determined using RATE-seq. (*A*) The relative fraction of reads mapping to the transcriptome increases with time, whereas the fraction of reads mapping to spike-ins decreases. (*B*) Representative example of RATE-seq data for a rapidly degraded gene *(CTK1*, purple) and a slowly degraded gene (*GIM4*, red). 95% CI for the estimated degradation rate constant are indicated by dashed lines. (*C*) The distribution of half-lives for all coding transcripts with the mean (red line) and median (blue line) half-life indicated.

We performed RATE-seq using replicate yeast populations growing in a defined rich medium during log phase. Following RNA-seq analysis, the relative counts (Supplemental Tables S2, S3) of spike-ins are observed to decrease with time and concomitantly, the proportion of counts mapping to the transcriptome increases (Fig. 2A). We found that the use of multiple spike-ins facilitated identification of technical biases potentially introduced during library preparation (Supplemental Fig. S8). The correlation of per transcript counts between replicates at the same time point is high (Spearman ρ = 0.98; Supplemental Fig. S9). To normalize transcript counts (Supplemental Table S4) we first determined the ratio of counts for each transcript to each spike-in, scaled each ratio, and then multiplied by the mean count of all spike-ins across all experiments to preserve the scale of the data (Materials and Methods). We studied the mean-variance relationship at each time point and found that the data are overdispersed (Supplemental Fig. S10). Therefore, to estimate the degradation rate constant for individual transcripts we performed a nonlinear weighted regression using normalized counts from the combined data set (Fig. 2B) (Materials and Methods). We determined confidence intervals for the estimated decay constant for each transcript using bootstrapped values from each time-point (Materials and Methods).

**FIGURE 3.**
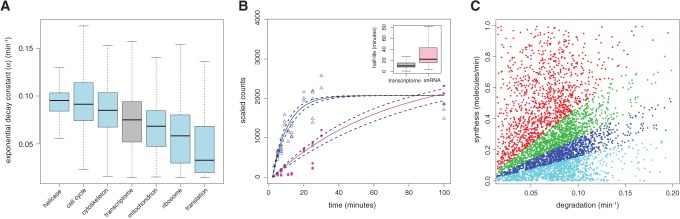
Coordinated regulation of mRNA abundance levels. (*A*) Decay rates for sets of functionally related genes defined by GO term categories are nonrandomly distributed. (*B*) RATE-seq can be used to estimate the kinetics of snRNAs (e.g., *snR48* [pink circles]) and lncRNAs (e.g., *ICR1* [blue triangles]). The boxplot (*inset*) displays the half-lives for snRNA and all other transcripts. (*C*) The global relationship between transcript synthesis and degradation in *S. cerevisiae*. The abundance of each mRNA is categorized into quartiles (first quartile: 0.001–1.58 mRNAs/cell [cyan], second quartile: 1.6–2.8 mRNAs/cell [blue], third quartile: 2.8–4.8 mRNAs/cell [green], fourth quartile: 4.8–66 mRNAs/cell [red]).

Using RATE-seq we determined degradation rate constants, and corresponding half-lives, with 95% confidence intervals (CI) for 5308 mRNAs (Supplemental Table S5). Most transcripts are rapidly degraded, with a mean and median half-life of 15 and 10 min, respectively (Fig. 2C). Thus, RATE-seq analysis estimates RNA half-lives that are shorter than most previous global estimates (Wang et al. 2002; Grigull et al. 2004; Miller et al. 2011; Munchel et al. 2011). Using bootstrapped values we find that for the majority of transcripts the estimated degradation rates have confidence intervals of ±20% (Supplemental Table S5). A previous study (Wang et al. 2002) showed that transcripts encoding functionally related gene products have similar decay rates. We find that genes within the same Gene Ontology (GO) terms also have similar decay rates (Supplemental Table S6) although the agreement between the estimated rates from the two studies is poor. Functional categories representing the most rapidly degraded transcripts include “Helicase activity” and “Regulation of cell cycle” whereas categories representing the most stable transcripts include “Cytoplasmic translation” and “Ribosome” (Fig. 3A).

In addition to variation in mRNA degradation rates we find evidence for variation in rates of noncoding transcripts including small nuclear RNA (snRNA) and long noncoding RNAs (lncRNA) (Fig. 3B). The population of snRNAs appear to be more stable than coding transcripts (Fig. 3B) and have similar half-lives, suggesting that the post-synthesis fate of snRNAs is coordinately regulated.

We compared mRNA half-lives estimated using RATE-seq to previously reported estimates in *Saccharomyces cerevisiae* (Supplemental Fig. S11). As noted in previous reports (Miller et al. 2011; Munchel et al. 2011), the agreement among mRNA half-lives using different methods is poor. Surprisingly, RATE-seq estimates correlate poorly with those reported using pulse-chase labeling with 4tU (Munchel et al. 2011). Our method has a number of differences that may account for this including the absence of poly(A) selection, the use of multiple spike-ins for normalization, and the use of untransformed data for nonlinear model fitting, which avoids errors introduced by linear transformation of data. In addition, mathematical modeling suggests that nucleotide recycling may slow the observed chase resulting in a systematic underestimation of mRNA decay rates (Supplemental Fig. S12). Our estimates are most similar to results obtained using DTA (Miller et al. 2011), which may reflect the fact that both methods isolate newly synthesized transcripts following label addition. Importantly, consistent with both existing in vivo labeling methods in budding yeast (Miller et al. 2011; Munchel et al. 2011), we find that the half-lives for ribosomal protein-coding genes is greater than the median half-life of all mRNAs (Supplemental Fig. S13). In contrast, in all studies using transcriptional inhibition ribosomal protein-coding transcripts are found to degrade as rapidly as the transcriptome average, which may reflect a stress response to the profound impact on cell physiology caused by these methods.

### The landscape of regulated transcript abundance

At steady state, transcript abundance levels are constant (i.e., *d*[RNA]/*dt* = 0) and transcript synthesis and degradation are related by the expression *k* = α[RNA]. Therefore, the rate of transcript production can be estimated using the degradation rate constant and the steady-state abundance of the transcript. As only a fraction of transcripts are labeled with 4tU (Fig. 1A), RATE-seq does not quantify RNA abundance. Therefore, we used published estimates of absolute transcript abundance from quantitative sequencing data (Lipson et al. 2009) to estimate rates of mRNA synthesis in steady-state conditions (Supplemental Table S5). Our estimates of mRNA synthesis rates are in good agreement with previous estimates using Genomic-Run-On (GRO) assays (Supplemental Fig. S14; Pelechano et al. 2010) with a linear correlation coefficient of *r* = 0.5. A source of discrepancy between the two data sets may be that our study estimates the rate of production of mature transcripts, whereas GRO estimates nascent transcription rates.

The combinatorial effect of variation in synthesis and degradation rates defines the landscape of regulated transcript abundance (Fig. 3C). Within this landscape it is clear that classes of transcripts defined by rapid synthesis and degradation have equivalent steady-state levels to classes of transcripts that are comparatively slowly synthesized and degraded. Understanding the sources and consequences of these different kinetics is central to understanding gene expression regulation.

## DISCUSSION

The abundance of a transcript is determined by both its rate of synthesis and its rate of degradation. To fully characterize the regulation of mRNA levels these rates must be uncoupled. Moreover, studying transcripts under their native control is critical as transcript stability may depend on *cis*-acting factors that associate with promoter regions (Bregman et al. 2011; Trcek et al. 2011).

RATE-seq is an efficient and general means of estimating transcriptome-wide absolute rates of RNA synthesis and degradation in steady-state conditions. In contrast to existing methods, it does not interfere with the cell's physiology, provides enhanced accuracy, obviates the potential impact of nucleotide recycling, and can be applied to a variety of types of transcripts on a genome-wide basis. In principle, incorporation of 4tU is feasible in all organisms using either endogenous or heterologous expression of UPRT (Cleary et al. 2005). Alternatively, 4sU can be used in organisms without endogenous nucleotide salvage pathways (Dölken et al. 2008). Therefore, we expect that RATE-seq will be of great utility for investigating the relationship between RNA synthesis and degradation in a variety of genotypes and organisms.

## MATERIALS AND METHODS

### Strains and growth conditions

Experiments were performed using either FY4 (*MATa*) or FY3 (*MATa ura3-52*), which are isogenic to S288C. All RATE-seq analyses were performed using the prototrophic strain FY4 in which a single colony was inoculated into an overnight culture in synthetic complete medium containing 500 µM uracil. The saturated overnight culture was back-diluted 1:50 into fresh medium of the same composition. Log phase cells were treated with 4tU to a final concentration of 500 µM. Cells were collected at multiple time points over the course of 100 min by vacuum filtration onto nitrocellulose filters and immediately frozen in liquid nitrogen.

### Synthesis of polyadenylated thiolated spike-in RNAs

To generate three RNA spike-ins with similar GC content to *S. cerevisiae* mRNAs, we cloned three different regions of the *Bacillus subtilis* genome. The three spike-ins (spike-in_700_, spike-in_900_, spike-in_1200_) have a GC content of 0.42 and lengths of 700, 900, and 1200 bases, respectively. Three regions of the *B. subtilis* genome were PCR amplified and cloned into the pSP64 poly(A) in vitro transcription vector (Promega). Plasmids were linearized using EcoRI restriction and run off transcription performed as recommended by the manufacturer with the addition of thiolated UTP:UTP at a ratio of 2:1 in the reaction. Spike-in RNA was subsequently treated with DNAse and purified.

### RNA extraction

RNA was purified from cells using a hot acid phenol/chloroform extraction. Briefly, 750 μL of lysis buffer (10 mM EDTA, 10 mM Tris, 0.5%SDS) was added to each sample and vortexed. An equal volume of acid phenol was then added to the sample and vortexed. Samples were incubated for 1 h at 65°C with occasional vortexing. Filters were removed and samples were placed on ice for 10 min. After centrifugation, the aqueous phase was transferred to Phase Lock Gel (PLG) tubes and an equal volume of chloroform added. The aqueous phase was collected and RNA was precipitated using two volumes of 95% ethanol and 0.1 volume of 3 M Sodium Acetate. RNA pellets were washed with 70% ethanol twice and dried at room temperature for half an hour and resuspended in RNAse free water.

### RNA biotinylation and streptavidin pull down

For biotinylation reactions 100 µg of total RNA was added to a solution of 10 mM Tris-HCl (pH 7.4), and 1 mM EDTA. Biotin-HPDP (1 mg/mL) was added to a final concentration of 2 µg for each 1 µg of RNA (Supplemental Fig. S15A). In addition, the three spike-in RNAs were pooled and 12 ng of the mixture added to the reaction mixture containing 100 µg of RNA sample. The reaction was allowed to proceed for 3 h in the dark, after which reactants were removed using chloroform extraction. RNA pellets were precipitated with 1 volume of isopropanol and 1/10 volume of 5 M NaCl. RNA pellets were washed once with 75% ethanol and resuspended in RNase-free water.

The biotinylated RNA was fractionated from unlabeled RNA using streptavidin magnetic beads (NEB) (Supplemental Fig. S15B). Pull downs were performed essentially as previously described (Zeiner et al. 2008). Beads were washed four times and then transcripts were cleaved from magnetic beads using β-mercaptoethanol (5%). RNA was precipitated with 1 volume isopropanol, 1/10 volume NaCl, and 3 µg of glycogen (Supplemental Fig. S15C).

### Dot blot analysis

For isolation of poly(A) RNA from total RNA, Oligo d(T)25 magnetic beads (New England Biosciences) were used in combination with a 12-tube magnetic rack. Beads were washed once in a binding buffer/wash buffer (20 mM Tris-HCl at pH 7.5, 500 mM LiCl, 1 mM EDTA) similar to manufacturer recommendations except that DTT was left out of the buffer, as this would cleave the RNA conjugated to biotin-HPDP. At least 40 µg of total RNA was added to 200 µL of beads. Samples were washed in 1x binding buffer, then 1x low-salt buffer, and eluted from beads in TE buffer following incubation for 3 min at 50°C.

For each sample, 200 ng of mRNA was blotted onto a Zeta-Probe nylon membrane (BioRad) using a BioRad DotBlot. The RNA was cross-linked using a UV cross-linker. The blot was blocked using blocking buffer (PBS, 10% SDS, 1 mM EDTA) for 20 min. Samples were then probed with Streptavidin Alkaline phosphatase in blocking solution (1:1000). The membrane was washed in PBS at decreasing concentrations of SDS (10%, 1%, 0.1%) for 10 min each. Spots were visualized using ECF substrate (GE Healthcare), visualized on a Typhoon FLA 9500, and analyzed using ImageQuant software.

### Depletion of ribosomal RNA

Following fractionation of thiolated transcripts, 100 ng was depleted of 18S and 25S ribosomal transcripts. Two rounds of ribosomal depletion were performed using LNA probes provided in the Ribominus kit (Invitrogen). RNA was then precipitated using 2 volumes ethanol, 1/10 volume 3 M sodium acetate, and glycogen. Pellets were resuspended in 6 µL of RNAse free water.

### Library preparation for Illumina sequencing

First strand synthesis of rRNA-depleted RNA was performed using the Super Script III kit (Life Technologies) and random priming using random hexamers. Second-strand synthesis was performed with dUTP in place of dTTP to enable strand-specific sequencing (Parkhomchuk et al. 2009). Samples were end repaired, A-tailed, and ligated to NEXTflex DNA Barcodes for multiplex sequencing. Adapter dimers were removed using AMPure beads (Agencourt). Samples were then treated with UNG and amplified using 10 cycles of PCR prior to sequencing. Samples were sequenced using an Illumina 2000 single-end 50-bp run.

### Sequence alignment

Ilumina sequencing reads were first filtered for rRNA sequences by aligning to the ribosomal DNA of the yeast genome using Bowtie with default settings. All remaining reads were then aligned to the rest of the yeast genome and the three spike-in sequences using Bowtie2 (Langmead et al. 2009). After converting SAM files to BAM files, reads were filtered based on quality scores of 20 or higher. The resulting BAM files were then used to calculate total counts per transcript using the *featureCounts* function of the *Subread* package in R, using the argument for strand specific counting. Each library had between 5 and 13 million reads mapping to non-rRNA transcripts.

### Data normalization

We performed RATE-seq using two biological replicates, with time points *k* = 3, 5, 7, 11, 13, and 25 min following label addition for replicate 1, and time points *k* = 5, 7, 9, 13, 20, 25, 30, and 100 min following label addition for replicate 2. To normalize data within each time series we used the following normalization scheme:

1) We first computed a ratio, *A*, between the read count *M*, for each gene *i* in replicate *j* at time point *k* and the read count *S* for each spike-in *n* in replicate *j* at time point *k*:

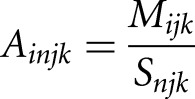

where *n* = 1,2,3 for each of the three spike-ins and *j* = 1 or 2 depending on the replicate
2) We then computed a scaling factor, β, for each spike-in by calculating the average ratio between each spike-in and a reference spike-in across all *K* time points within a replicate *j*:

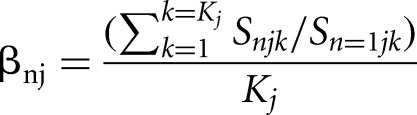

3) To normalize the data within a replicate, *j* we multiplied the ratio, *A* for each gene by the scaling factor:

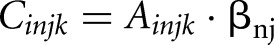

4) To return the data to the original scale we then multiplied the normalized ratio for each gene by the average spike-in count across all *K* time points from both replicates:

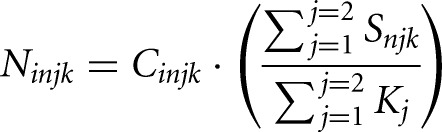

We excluded all data from a time-point if a spike-in was deviant in its expected behavior. Thus, each time point has between two and six values depending on whether the time point was replicated and whether any data were removed.

### Model fitting

The approach to equilibrium method assumes transcript decay follows first order kinetics and transcript synthesis follows zeroth order kinetics. This leads to the following two equations:

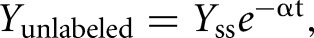
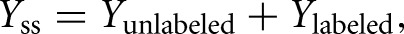

where *Y*_unlabeled_ is the abundance of unlabeled transcript, *Y*_labeled_ is the abundance of labeled transcript, *Y*_ss_ is the total abundance of a transcript, and α is the transcript's degradation rate constant. The approach to equilibrium equation is then obtained by substitution of equation 1 for the value of *Y*_unlabeled_ in equation 2 and solving for *Y*_labeled_ leading to 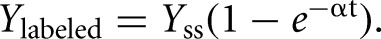


Based on this equation, we modeled the abundance of labeled transcript for each mRNA as 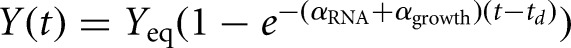, where *Y*(*t*) is the amount of the labeled transcript at time *t*, *Y*_eq_ is the abundance of labeled transcript at steady state, α_RNA_ is the transcript's degradation rate constant, α_growth_ is the growth rate constant of the culture, *t* is the time after addition of label, and *t*_*d*_ is a time delay between the addition of label and the time at which labeled transcripts can be detected.

To calculate the degradation rate constant for each transcript, we performed nonlinear regression, estimating both α (the summation of α_RNA_ and α_growth_) and *Y*_eq_. To account for biological variation, we combined the data from both replicates to generate a single parameter estimate for α_RNA_ and *Y*_eq_. Because the variance of the RATE-seq data increases with increasing time we used weighted least squares regression with weights of 1/Y, which avoids undue influence of later time points on the model fit, using the *gnls* function in the *nlme* package in R. To minimize the parameters that we needed to estimate we set the time delay parameter, *t*_*d*_ equal to 2 min, since labeled transcripts were pulled down as early as 3 min after addition of 4tU. As the doubling time of the culture is 100 min, α_growth_ is equal to 0.0069. Transcript half-lives were calculated as ln(2)/(α-0.0069).

We calculated 95% confidence intervals of the estimated degradation rate constant by randomly sampling with replacement the equivalent number of points from normalized data for each mRNA 1000 times. When resampling fails to sample timepoints toward the latter part of the curve we found that the nonlinear regression frequently failed to converge. Therefore, we used bootstrapped values to estimate only α keeping *Y*_eq_ the same for all iterations.

The data and model fit for each gene can be visualized using the available R script *rateSeqFit.R* using the function curve.generator().

### Assessment of labeling efficiency and bias

We estimated the amount of 4tU labeling using a colorimetric Dot Blot analysis of labeled RNA and a synthesized oligonucleotide containing a 5′ biotin label. A standard curve was generated by diluting known quantities of the labeled oligonucleotide and used to estimate the number of labels in an RNA sample of known mass. Assuming an average transcript length of 1200 nucleotides we estimate that approximately one out of 500 uracil is labeled after 35 min of labeling under our conditions.

To test whether 4tU is preferentially incorporated, we performed a DNA microarray analysis of 4tU labeled RNA compared with the unfractionated sample (Supplemental Fig. S16). Consistent with previous observations (Miller et al. 2009), there is a slight dependency of label incorporation on length. Thus, RNA-seq analysis of 4tU labeled transcripts is expected to result in increased counts for longer transcripts as a result of both increased labeling efficiency and the larger target size of longer transcripts (Supplemental Fig. S17). This labeling bias affects the steady-state equilibrium value for each transcript (Supplemental Fig. S18). However, this bias does not affect the estimate of the decay rate as there is no relationship between the counts of labeled transcripts at any time point and our estimate of the decay rate constant (Supplemental Fig. S19).

### Estimation of mRNA synthesis rates

To estimate the synthesis rate for each transcript we assumed that the rate of change in mRNA (*d*RNA/*dt*) at steady-state is equal to zero and therefore used the relationship *k =*α[mRNA]. We also assumed 60,000 mRNA/cell (Zenklusen et al. 2008). Confidence intervals for mRNA synthesis rates were calculated using the 95% CI values determined for α_RNA_.

### Gene enrichment analysis

Gene enrichment analysis was performed as in (Gresham et al. 2010). Nonrandom distribution of decay rates for each GO SLIM category as compared with the genes not in the category were identified using the Wilcoxon-Mann-Whitney test in R.

### R functions and packages

All analyses were performed using R (R Core Team 2013) and several open source packages. The functions *featureCounts* of the *Subread* package and *gnls* of the *nlme* package were used for data analysis of nonlinear regression. The following functions and packages, in addition to base functions in R and custom written functions were used for presentation of figures: *subplot* of the *TeachingDemos* package, *axis.break* of *Plotrix*, and *heatscatter* of *LSD*.

### Accession codes

Sequencing data are available through the Sequence Read Archive under BioProject ID PRJNA236614.

## SUPPLEMENTAL MATERIAL

Supplemental material is available for this article.

## Supplementary Material

Supplemental Material
