# Supplementary material for: Determination of in vivo RNA kinetics using RATE-seq
Source: RNA. 2014 Oct;20(10):1645–52. doi: 10.1261/rna.045104.114 (PMC4174445; doi:10.1261/rna.045104.114)
Supplement: Supplemental Material [file supp_20_10_1645__index.html]

Determination of in vivo RNA kinetics using RATE-seq — Determination of in vivo RNA kinetics using RATE-seq — Supplemental Material 

# Determination of in vivo RNA kinetics using RATE-seq

## Supplemental Material

**Files in this Data Supplement:**

- Supp Fig S10.pdf
- Supp Fig S11.pdf
- Supp Fig S12.pdf
- Supp Fig S13.pdf
- Supp Fig S14.pdf
- Supp Fig S16.pdf
- Supp Fig S17.pdf
- Supp Fig S18.pdf
- Supp Fig S19.pdf
- Supp Fig S2.pdf
- Supp Table S1.pdf
- Supp Fig S1.pdf
- Supp Fig S3.pdf
- Supp Fig S5.pdf
- Supp Fig S15.pdf
- Supp Fig S4.pdf
- Supp Fig S6.pdf
- Supp Fig S7.pdf
- Supp Fig S8.pdf
- Supp Fig S9.pdf
- Supp Table S5.xls
- Supp Table S7.xls
- Supp Legends.docx
- Supp readme\_Table.txt
- Supp Table S4.xls
- Supp Table S6.xls
- Supp Table S2.xls
- Supp rateSeqFit.R
- Supp Table S3.xls
