# Supplementary figures and images for: Determination of in vivo RNA kinetics using RATE-seq
Source: RNA. 2014 Oct;20(10):1645–52. doi: 10.1261/rna.045104.114 (PMC4174445; doi:10.1261/rna.045104.114)

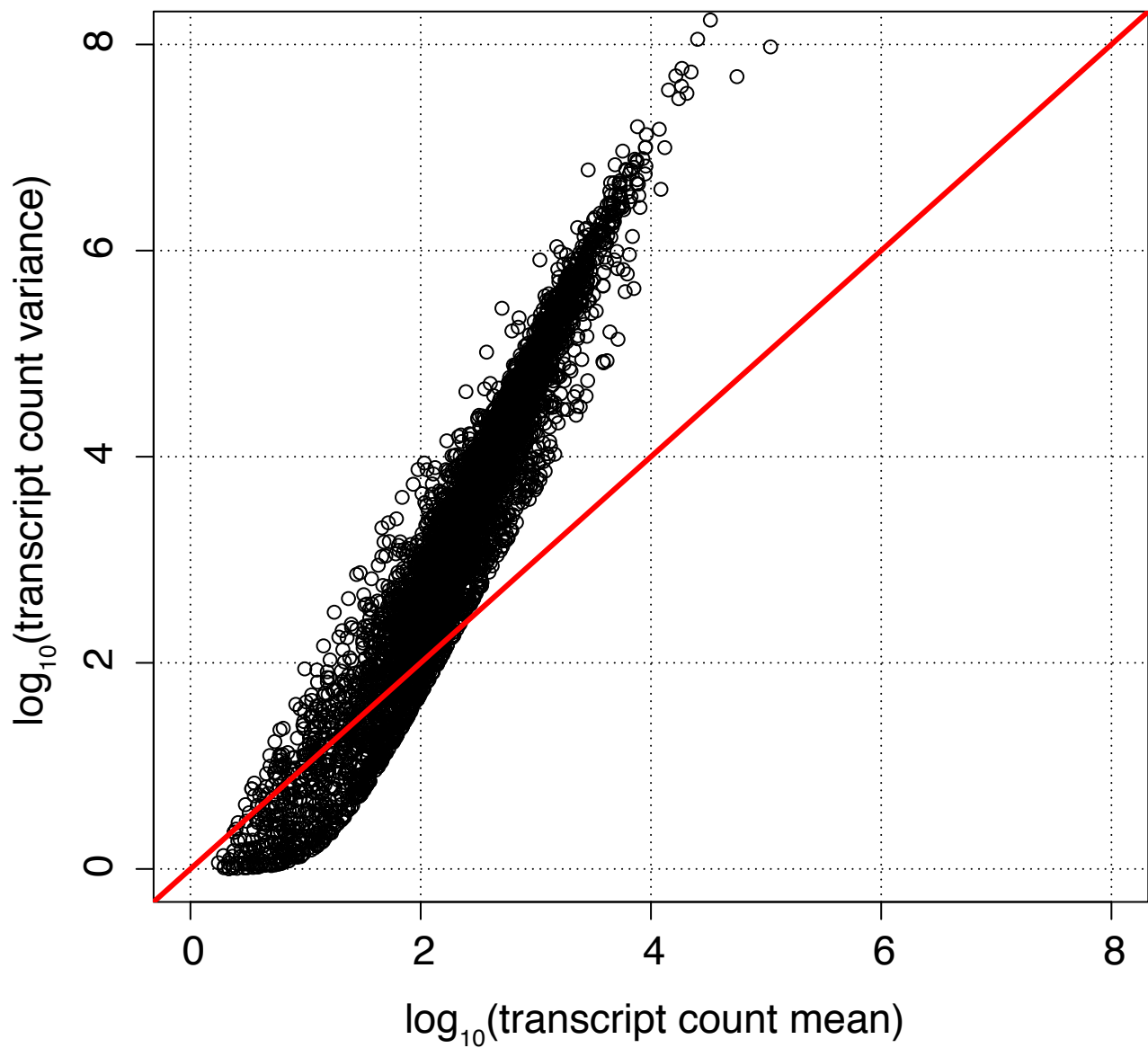

Supplement: Supplemental Material [file supp_045104.114_Figure_S10.pdf]

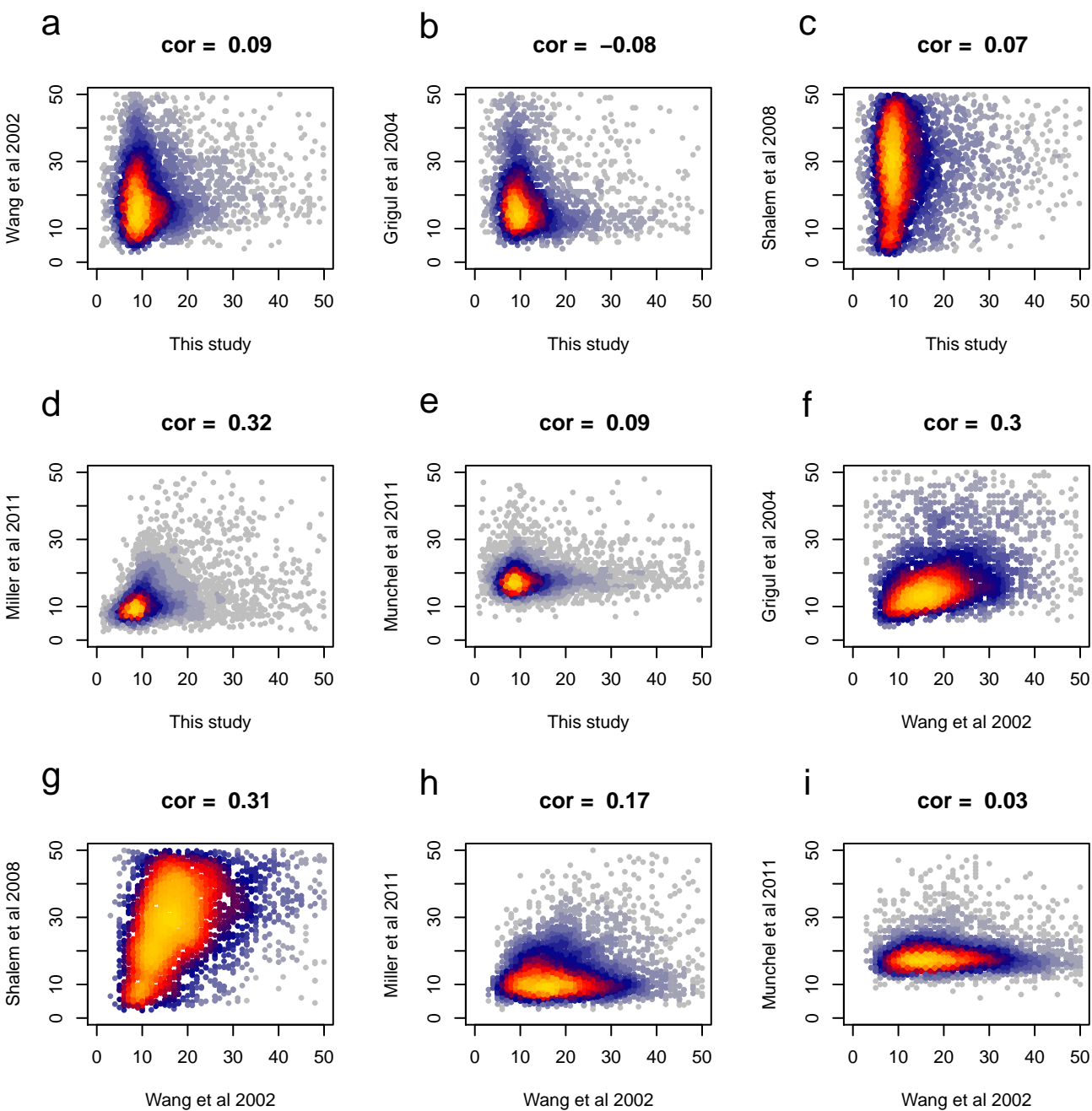

Supplement: Supplemental Material [file supp_045104.114_Figure_S11.pdf]

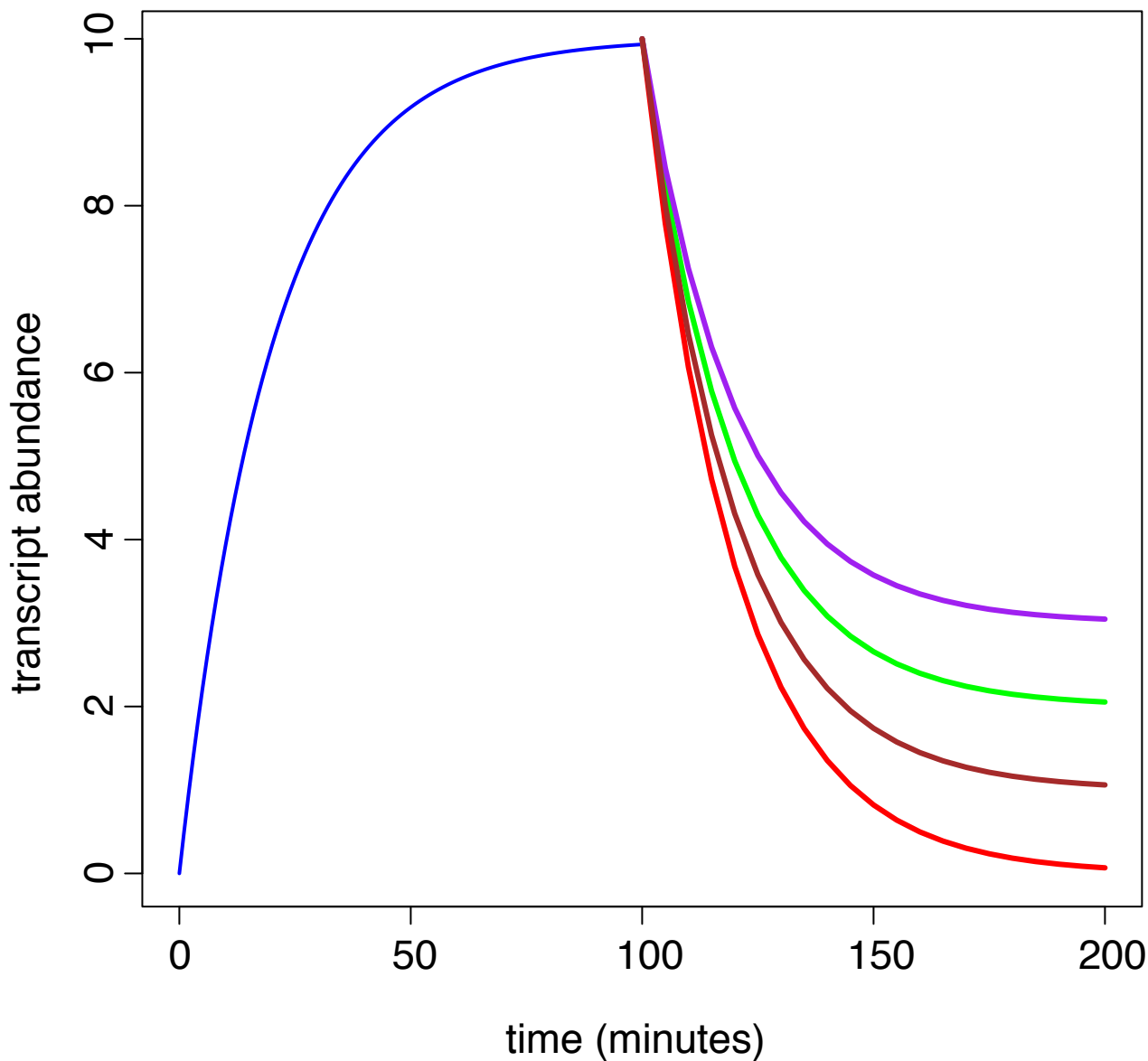

Supplement: Supplemental Material [file supp_045104.114_Figure_S12.pdf]

**a**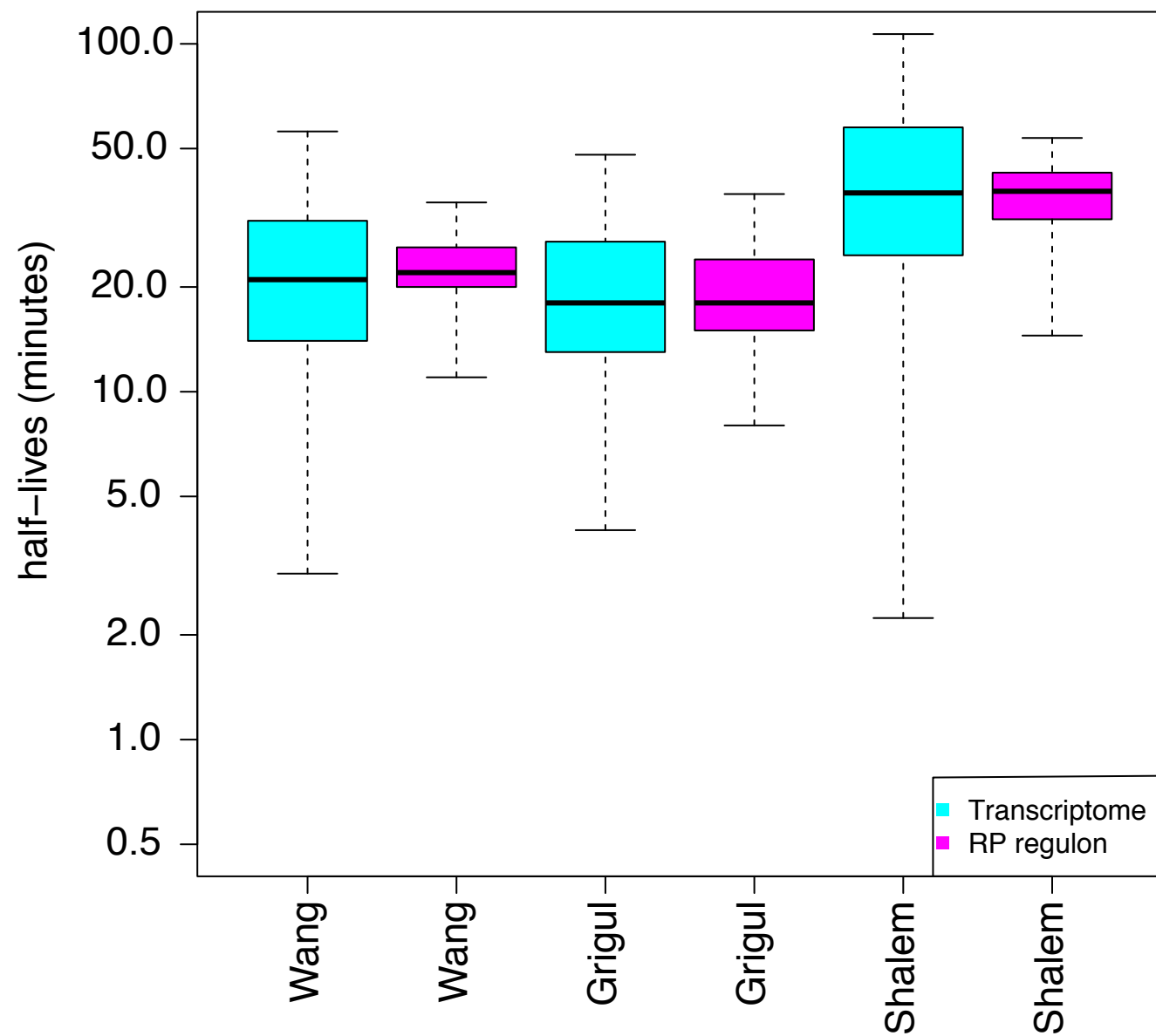**b**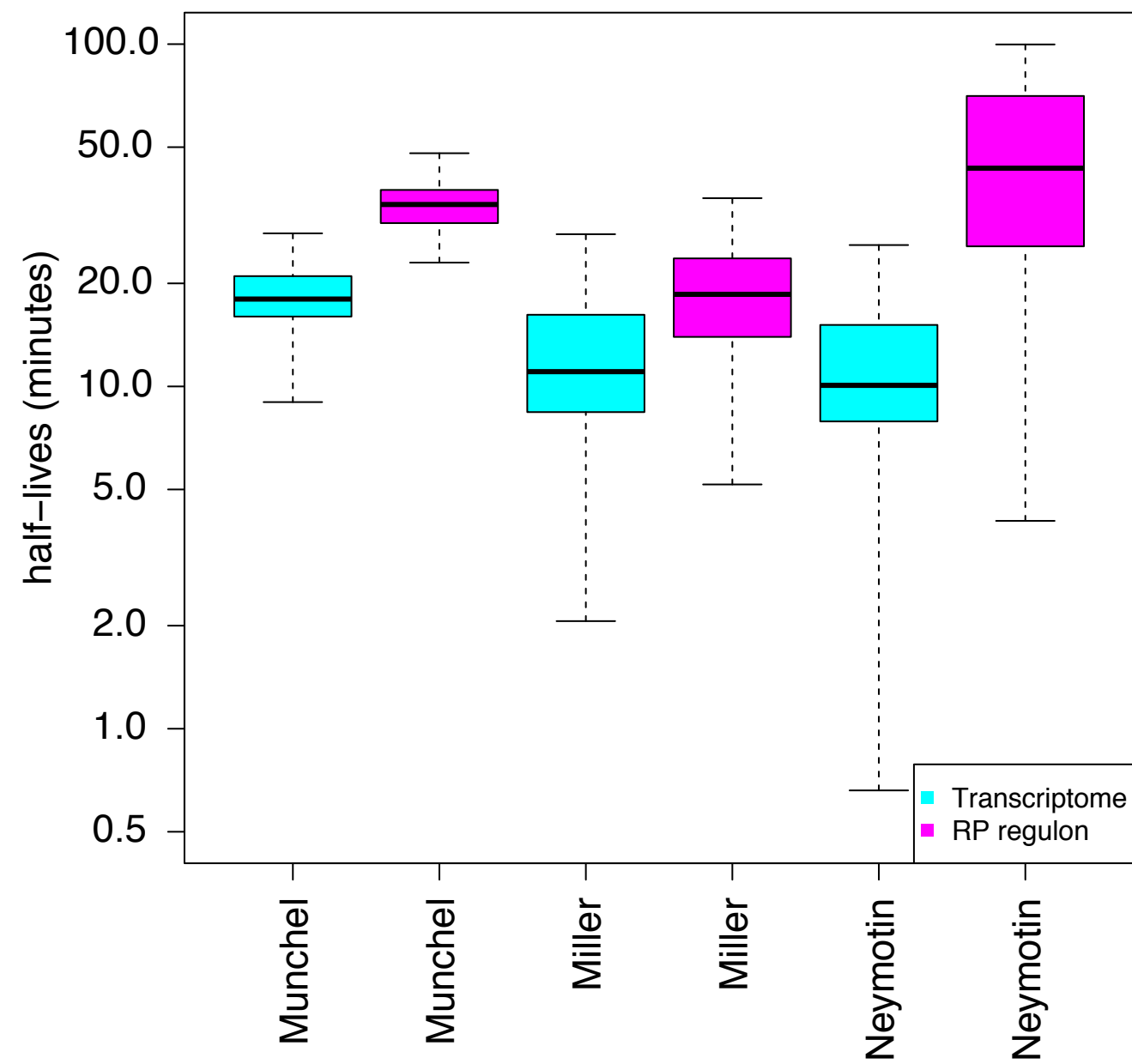

Supplement: Supplemental Material [file supp_045104.114_Figure_S13.pdf]

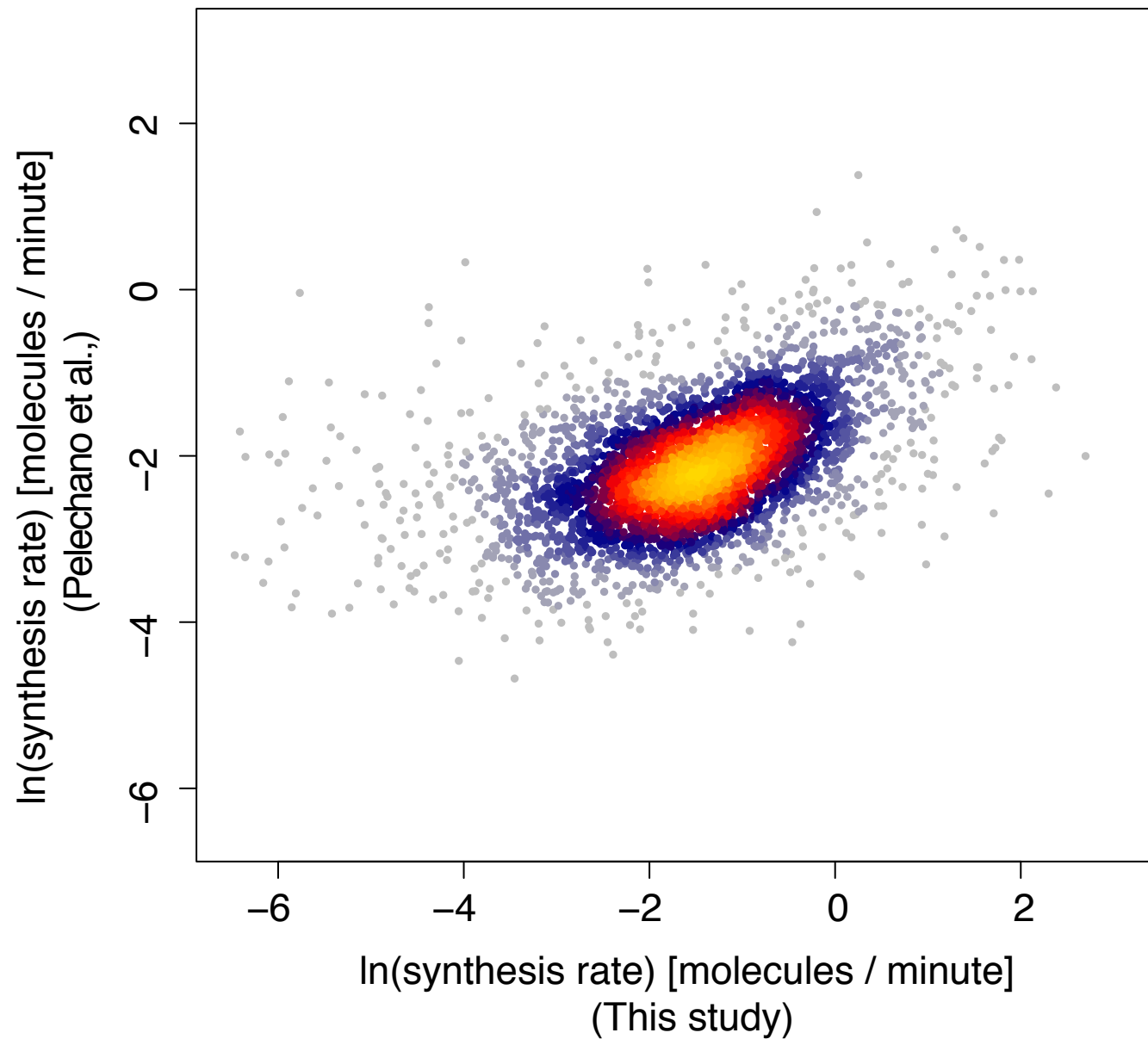

Supplement: Supplemental Material [file supp_045104.114_Figure_S14.pdf]

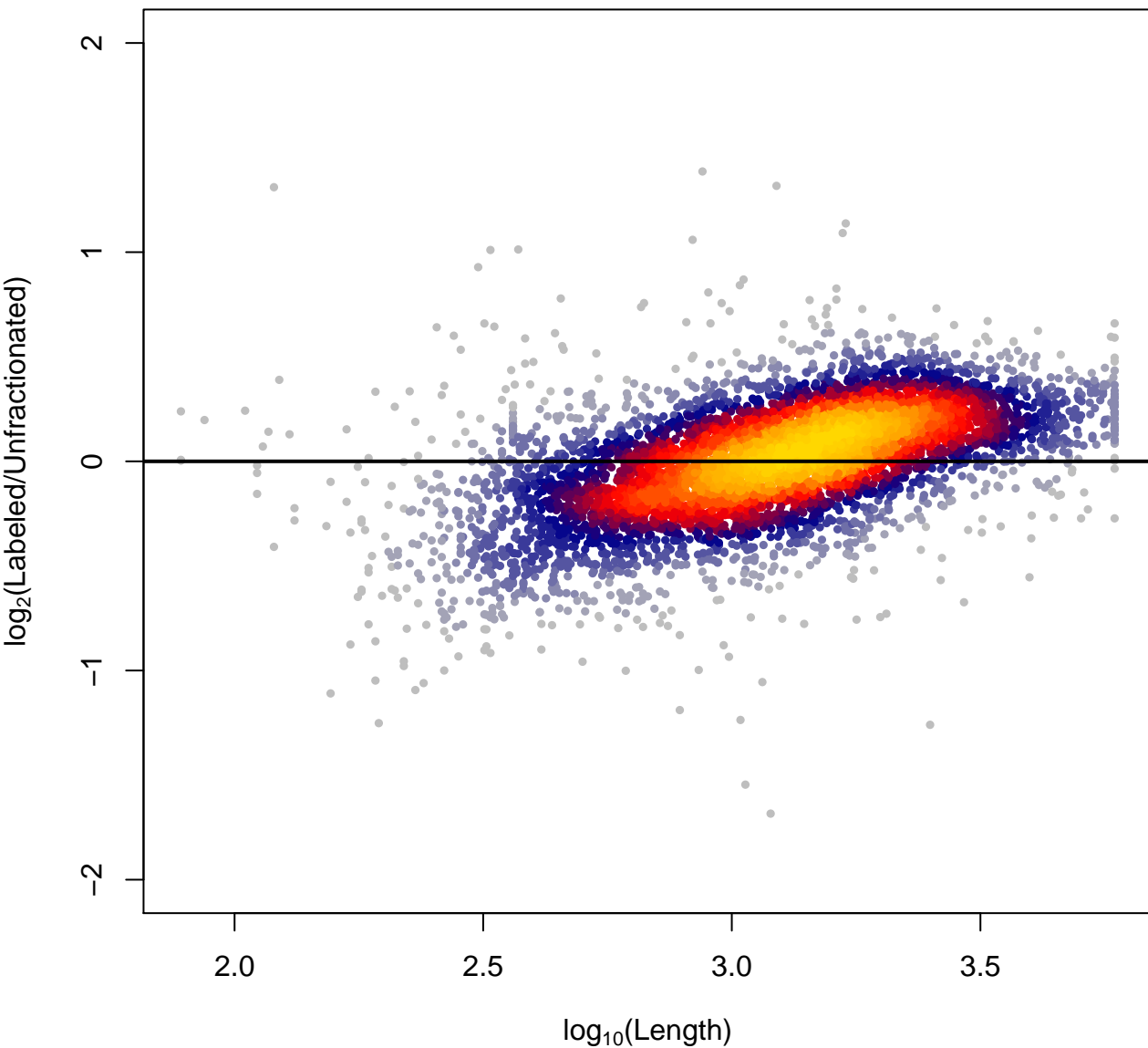

Supplement: Supplemental Material [file supp_045104.114_Figure_S16.pdf]

**a****t=5min**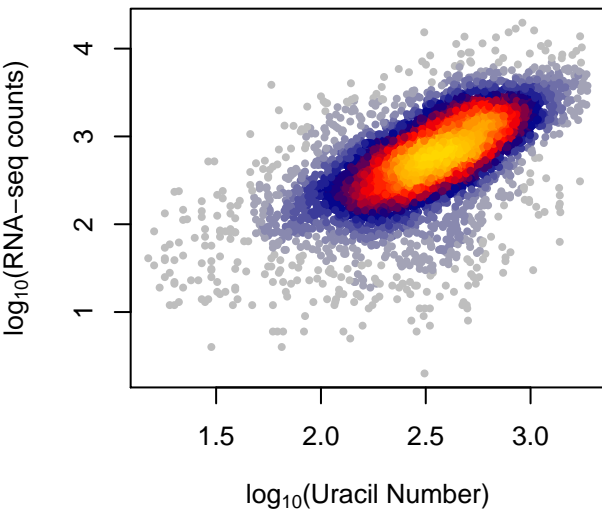**b****t=13min**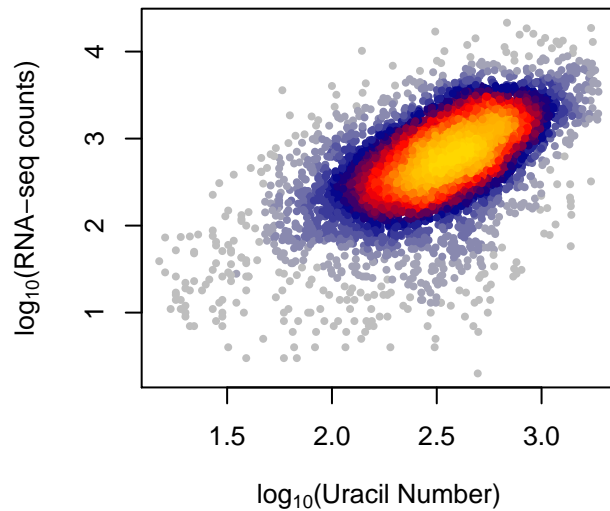**c****t=25min**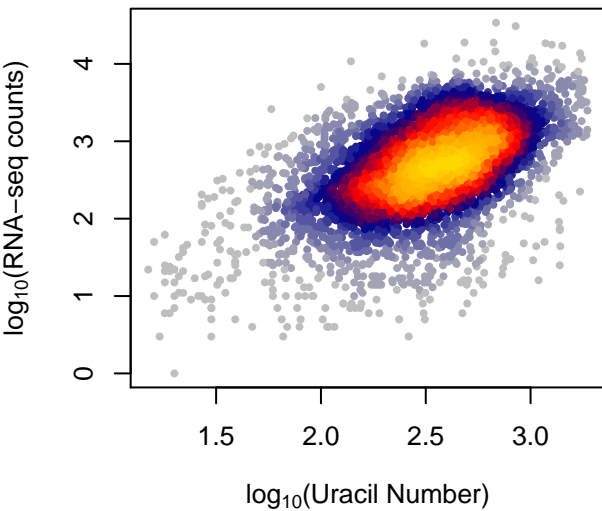**d****t=100min**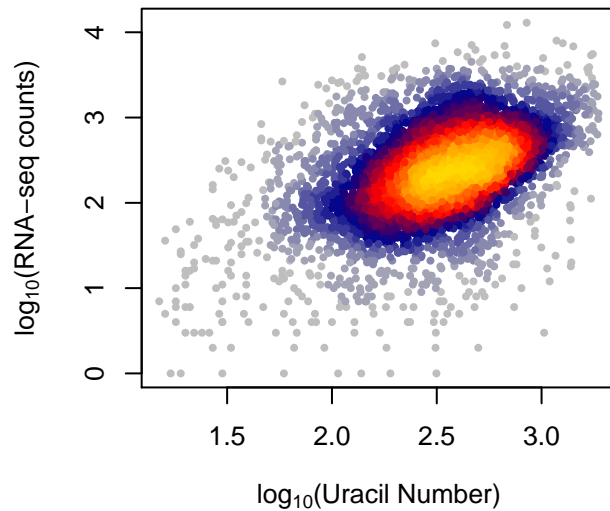

Supplement: Supplemental Material [file supp_045104.114_Figure_S17.pdf]

**a** **t=5min**

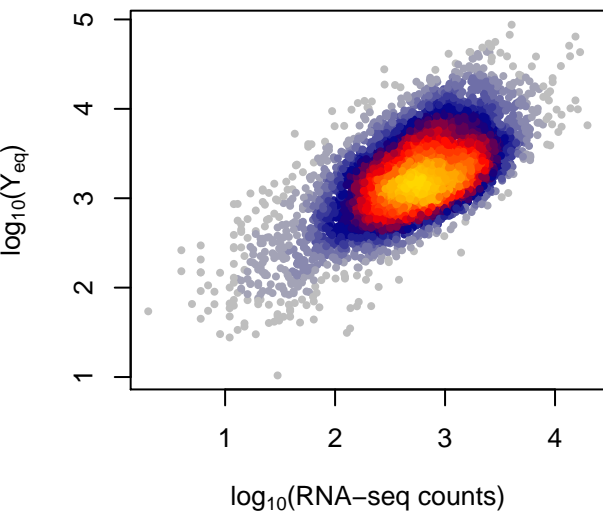

**b** **t=13min**

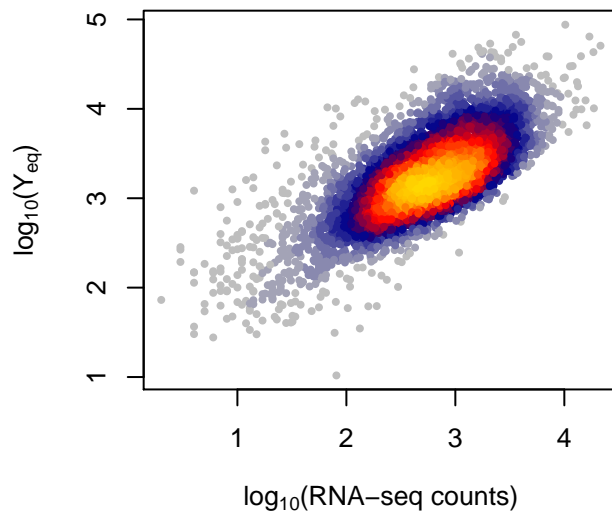

**c** **t=25min**

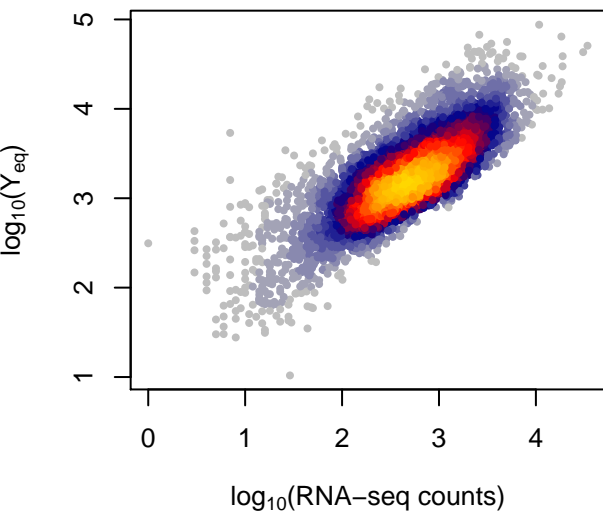

**d** **t=100min**

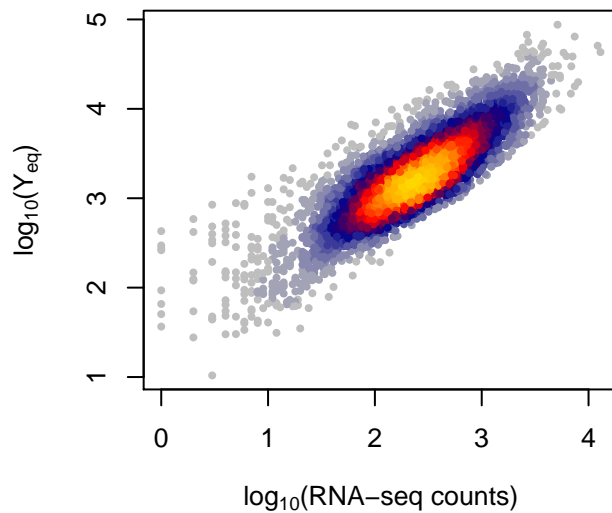

Supplement: Supplemental Material [file supp_045104.114_Figure_S18.pdf]

**a** **t=5min**

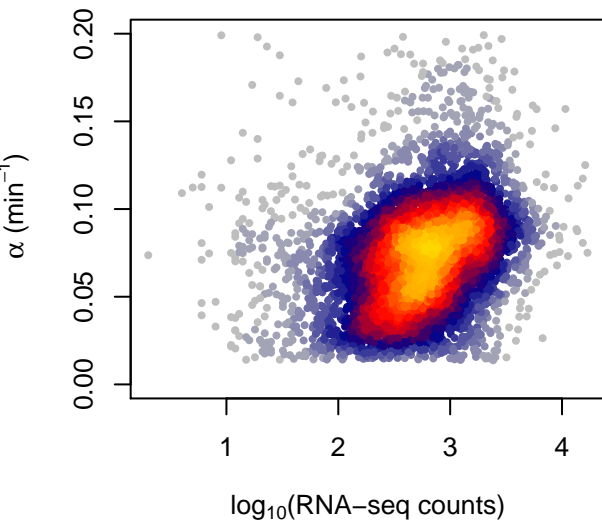

**b** **t=13min**

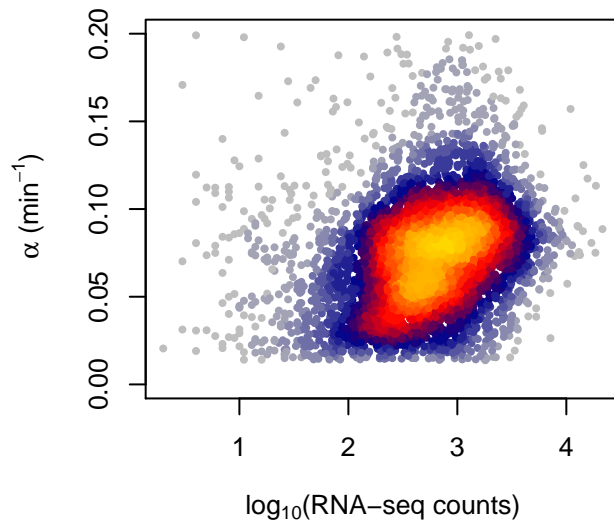

**c** **t=25min**

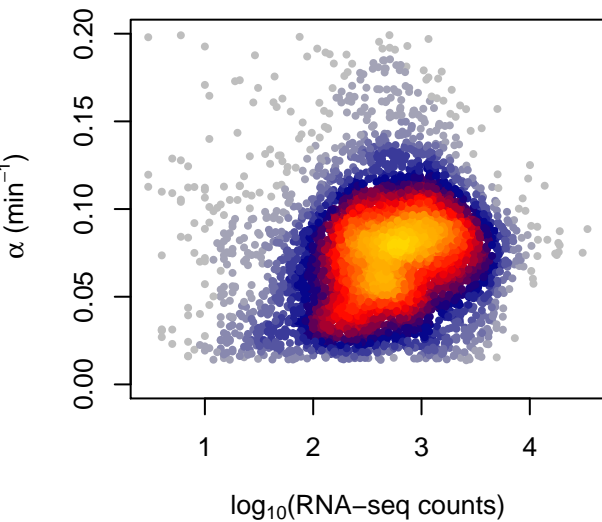

**d** **t=100min**

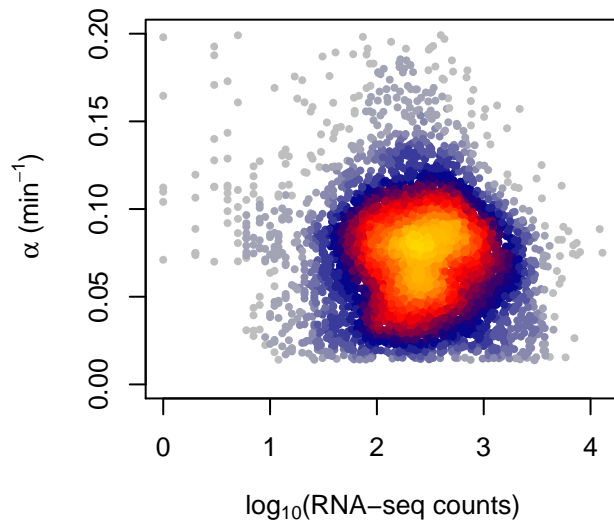

Supplement: Supplemental Material [file supp_045104.114_Figure_S19.pdf]

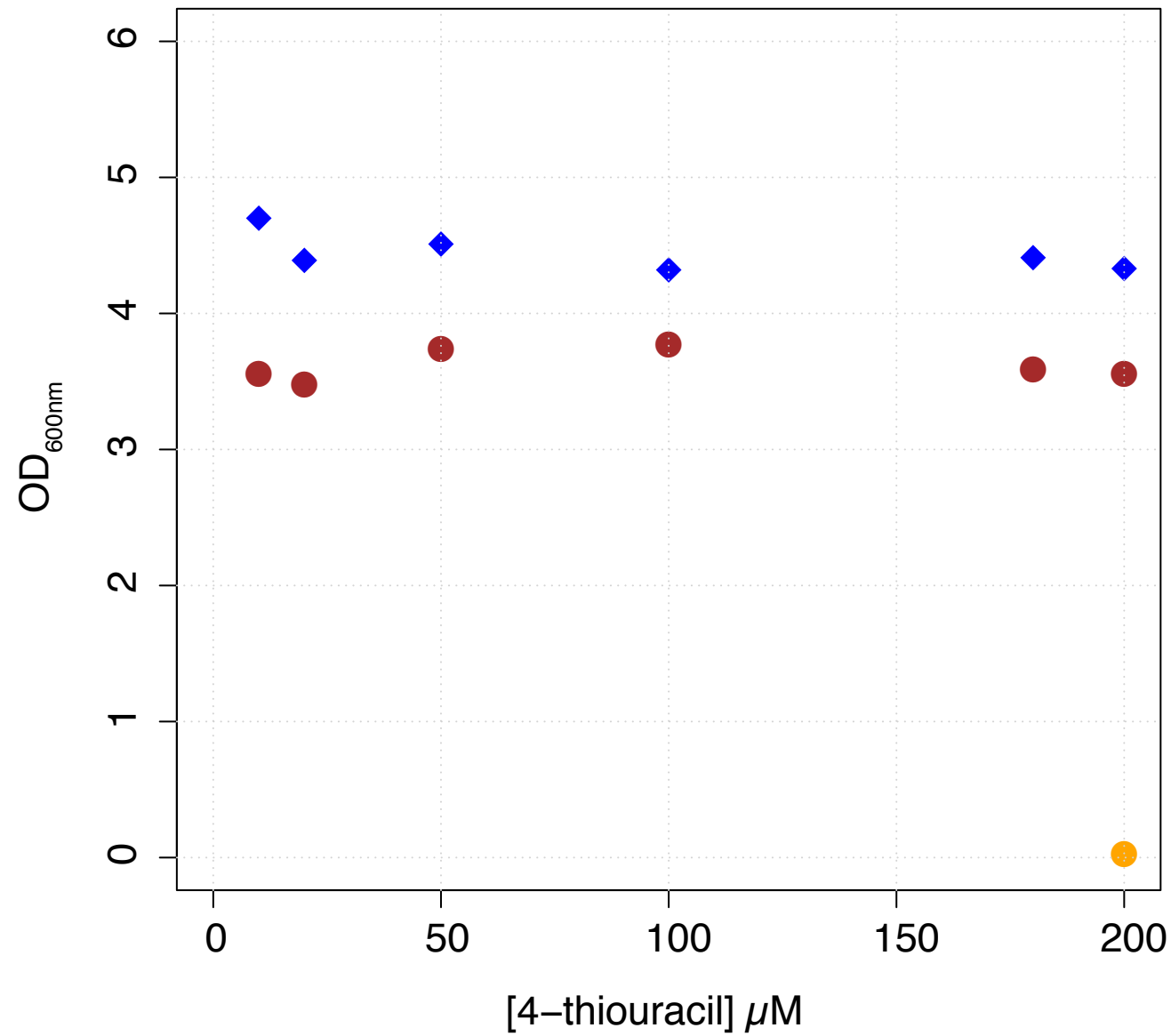

Supplement: Supplemental Material [file supp_045104.114_Figure_S2.pdf]

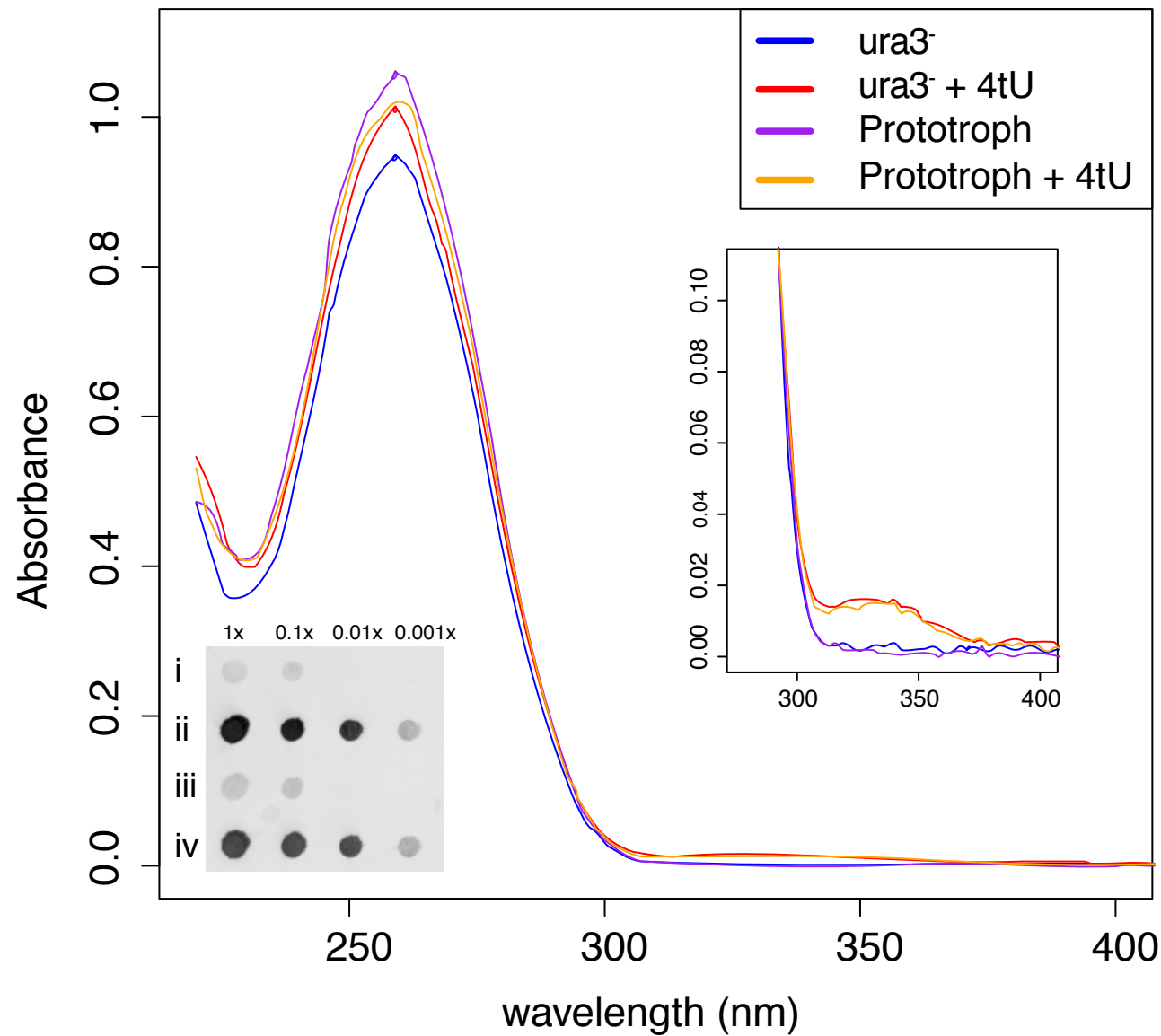

Supplement: Supplemental Material [file supp_045104.114_REVFigure_S1.pdf]

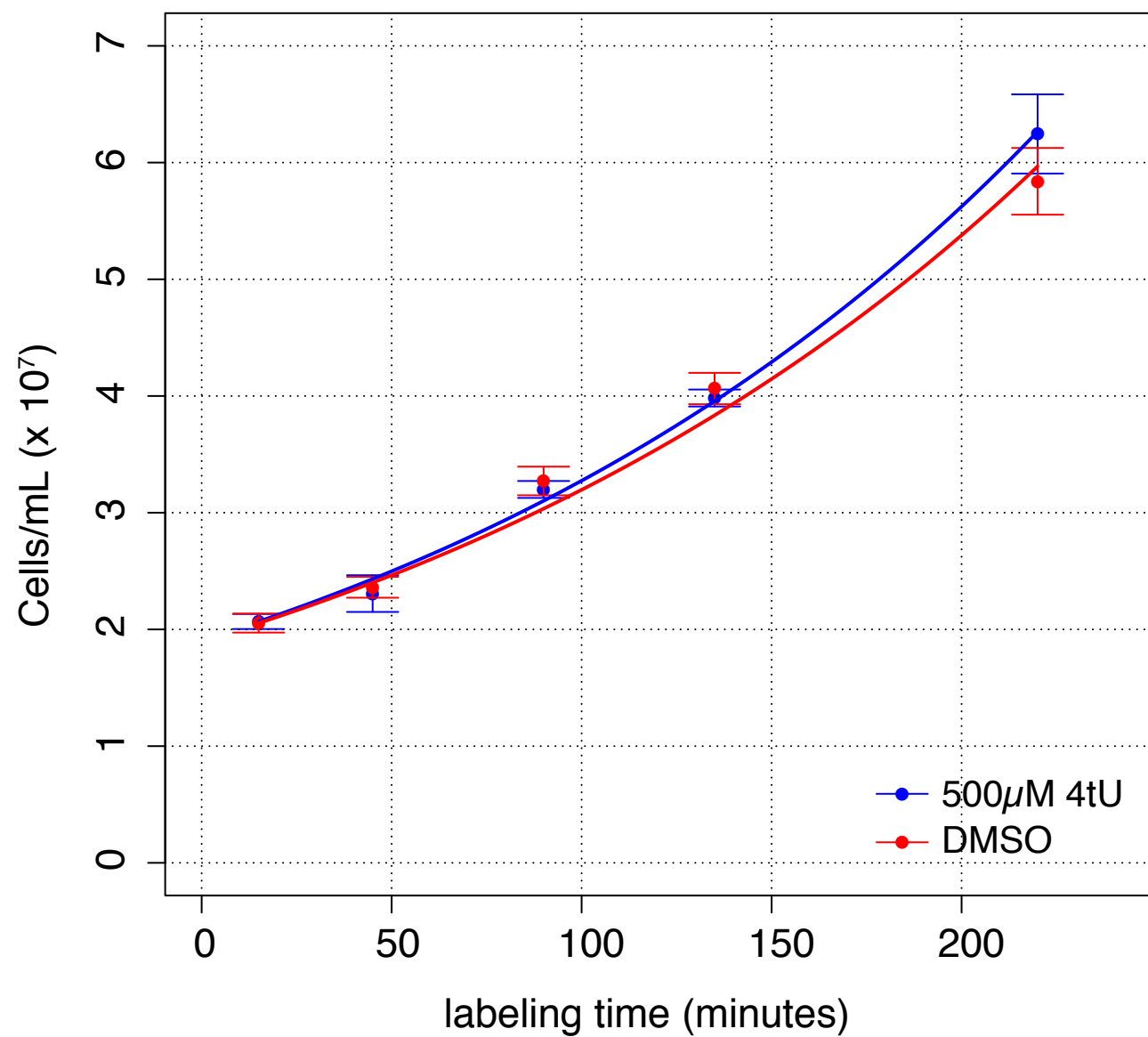

Supplement: Supplemental Material [file supp_045104.114_REVFigure_S3.pdf]

**a**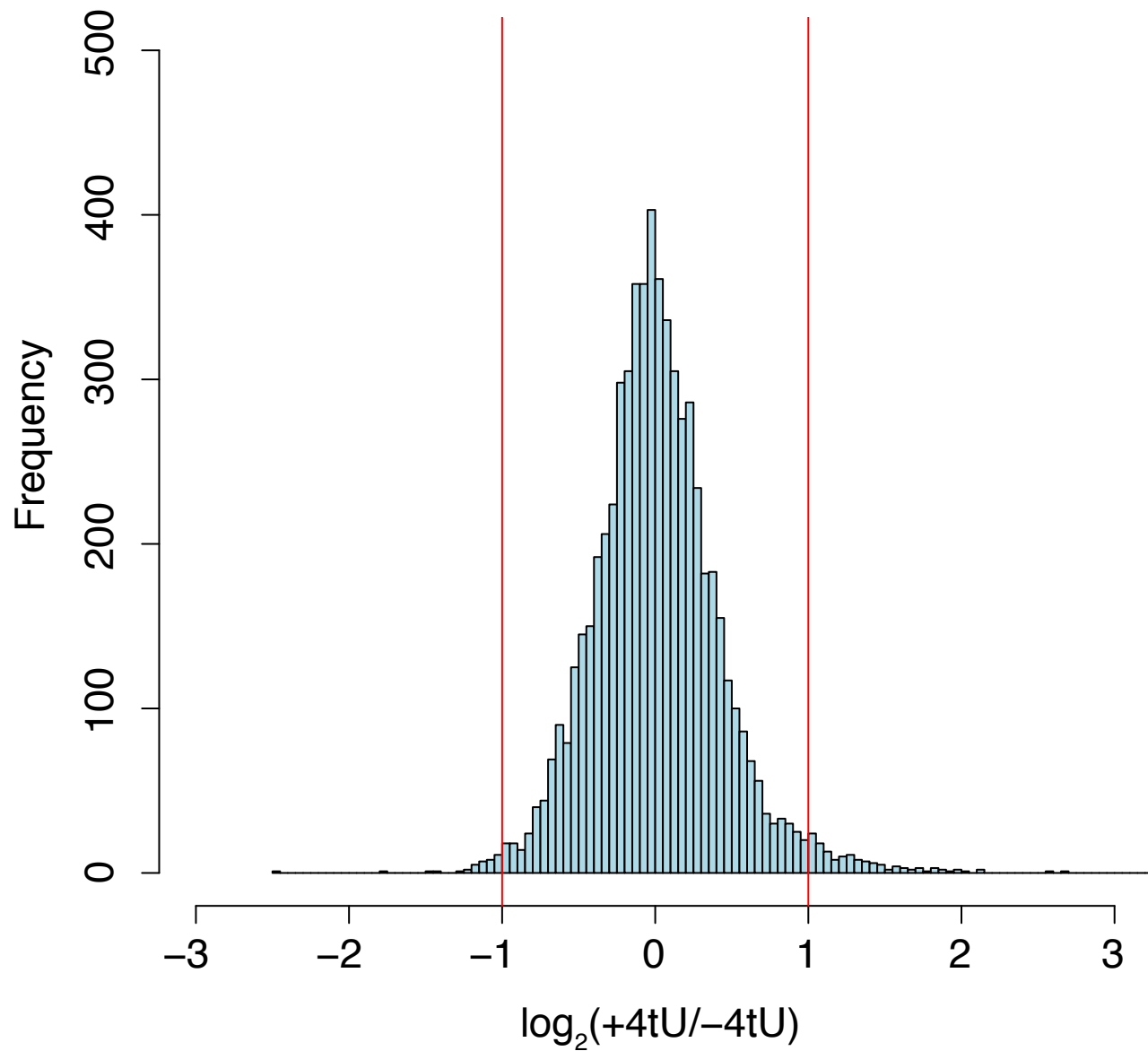**b**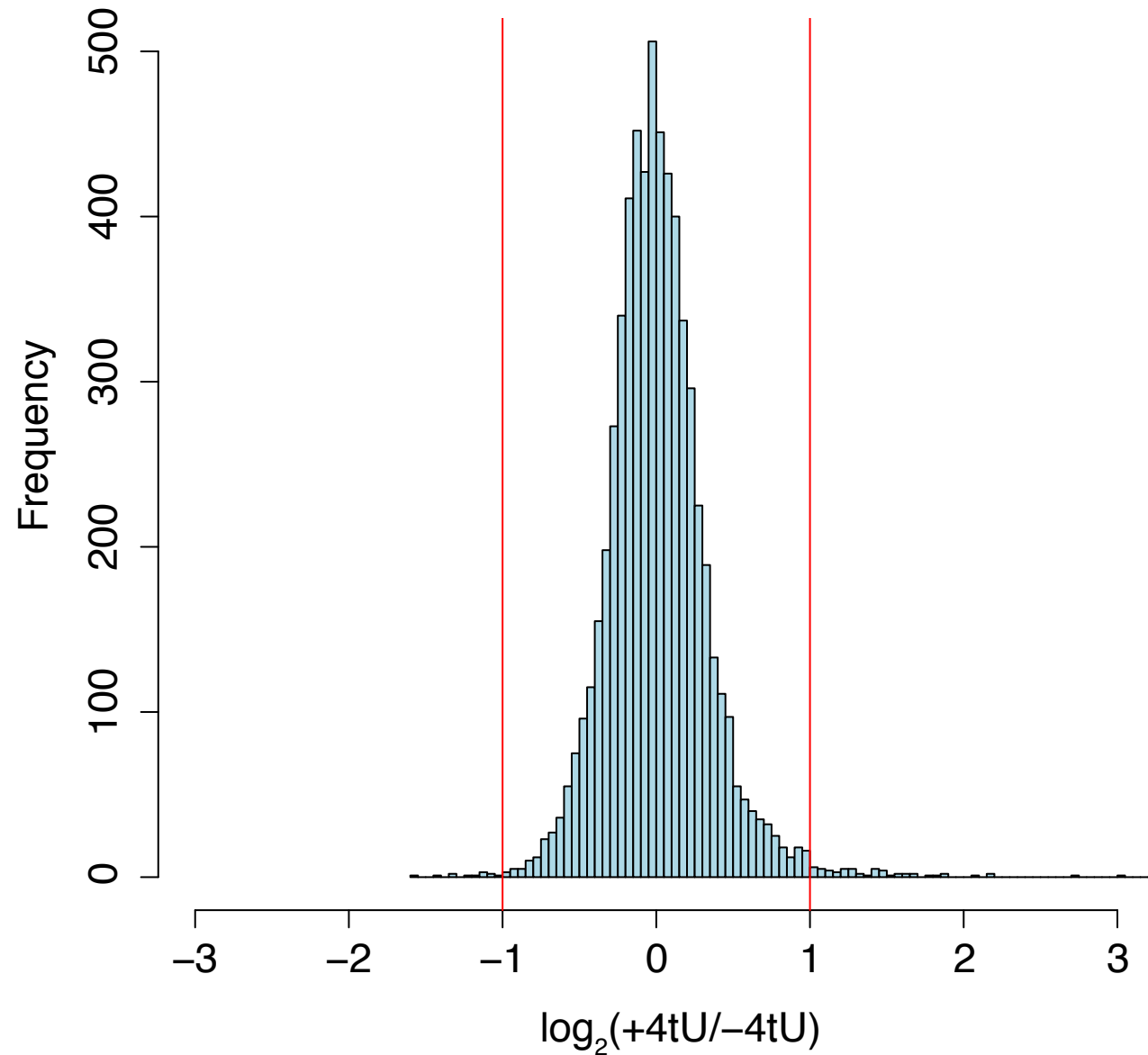

Supplement: Supplemental Material [file supp_045104.114_REVFigure_S5.pdf]

**a**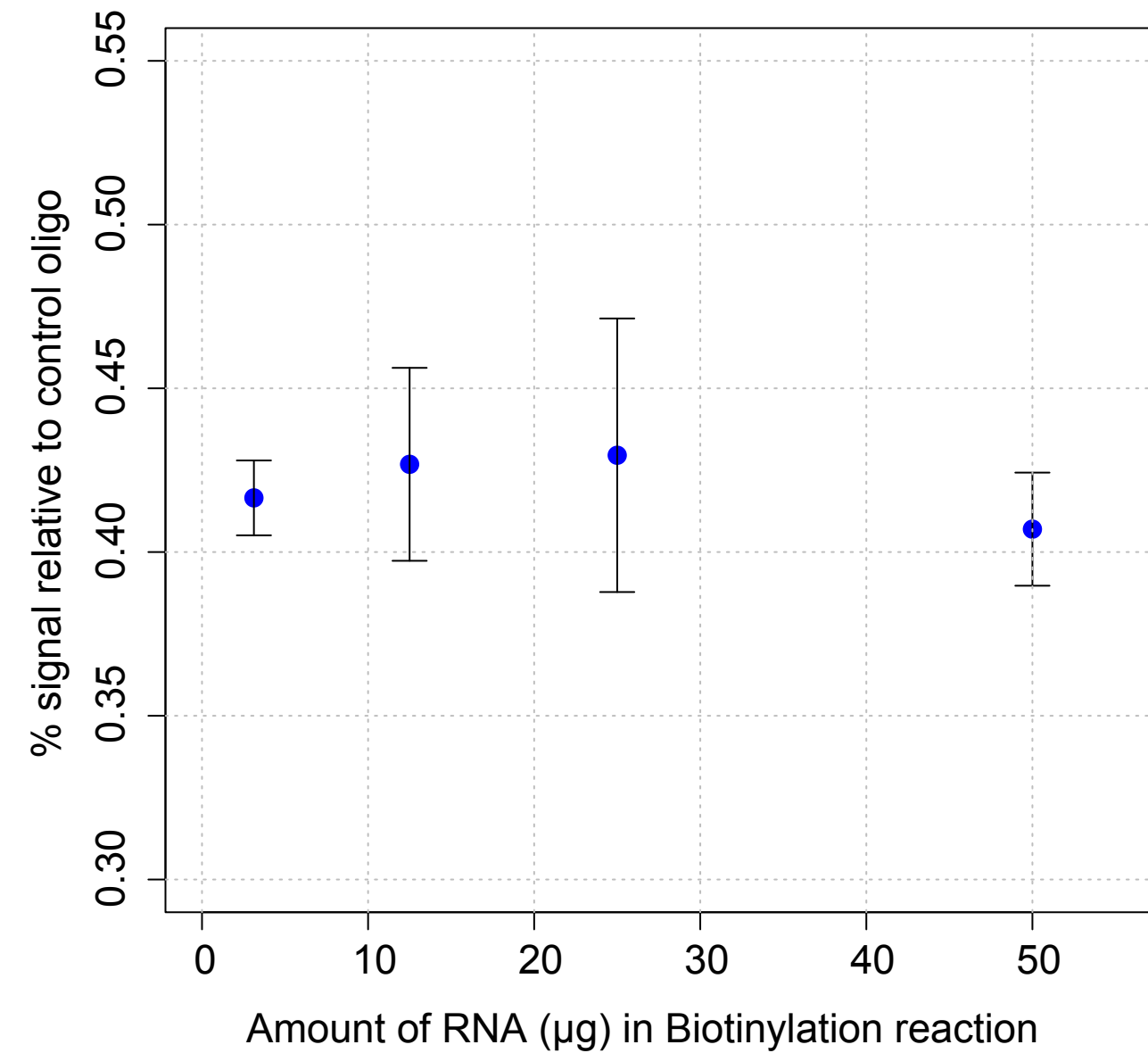**b**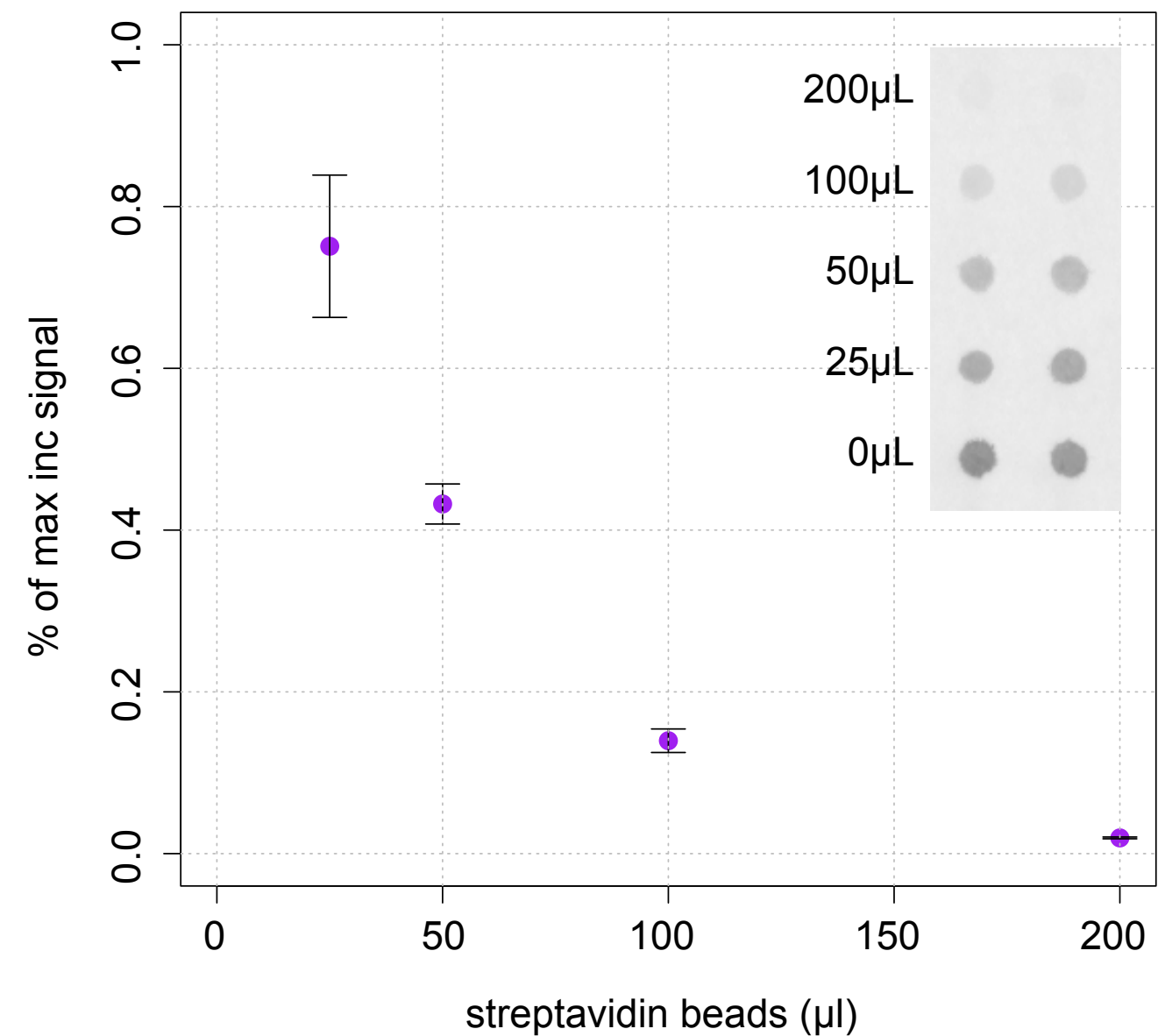**c**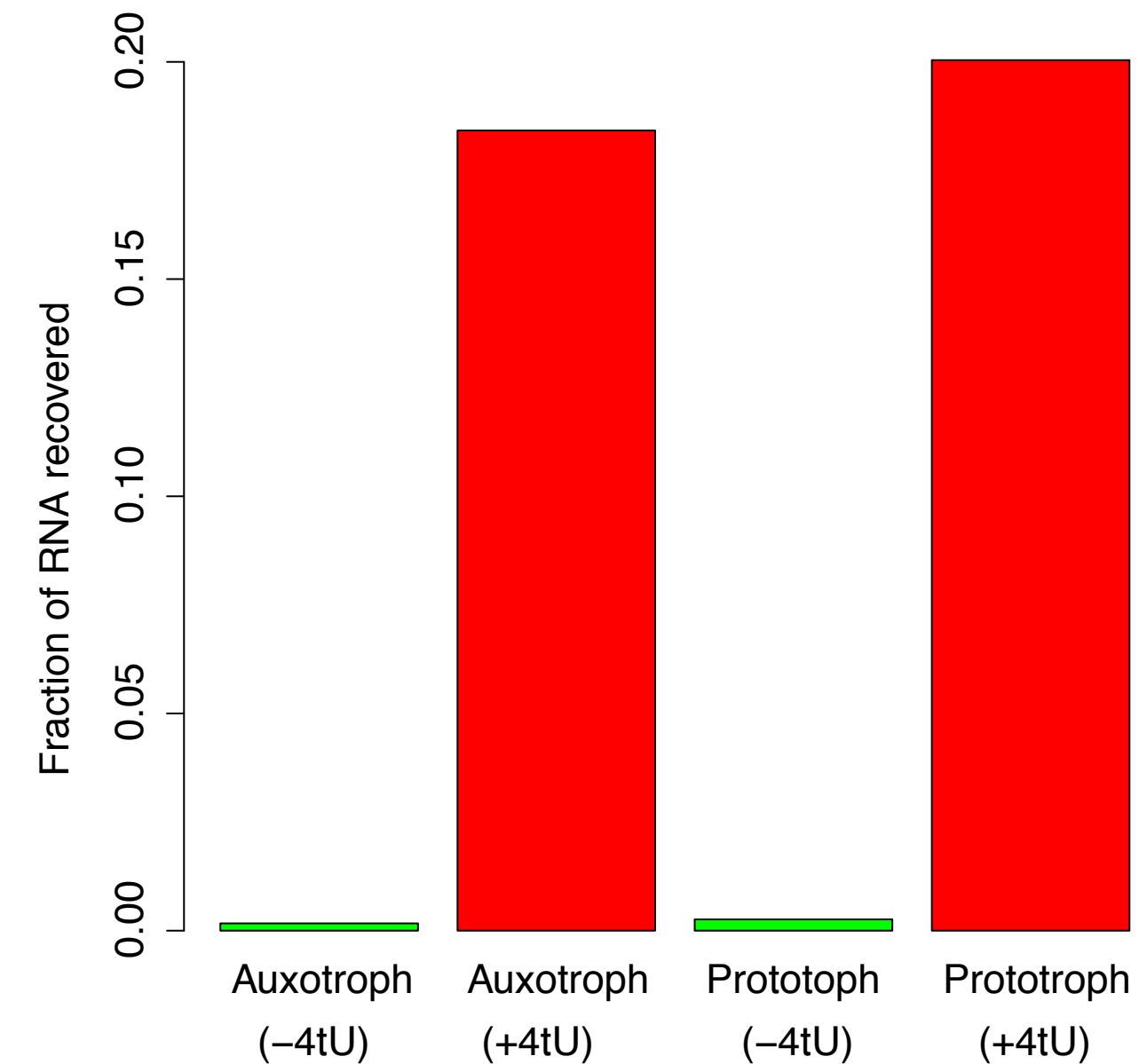

Supplement: Supplemental Material [file supp_045104.114_REVFigure_S15.pdf]

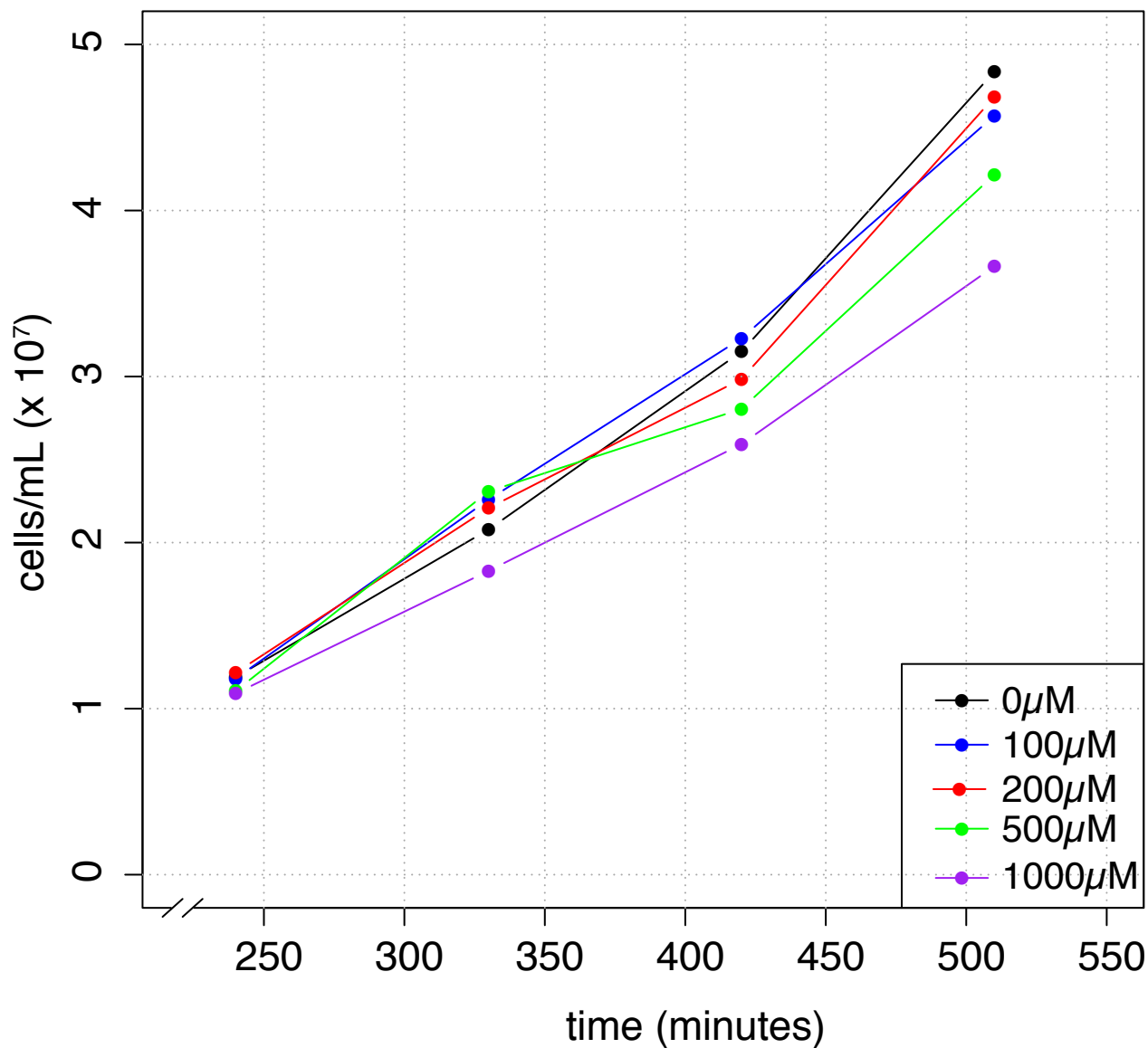

Supplement: Supplemental Material [file supp_045104.114_Figure_S4.pdf]

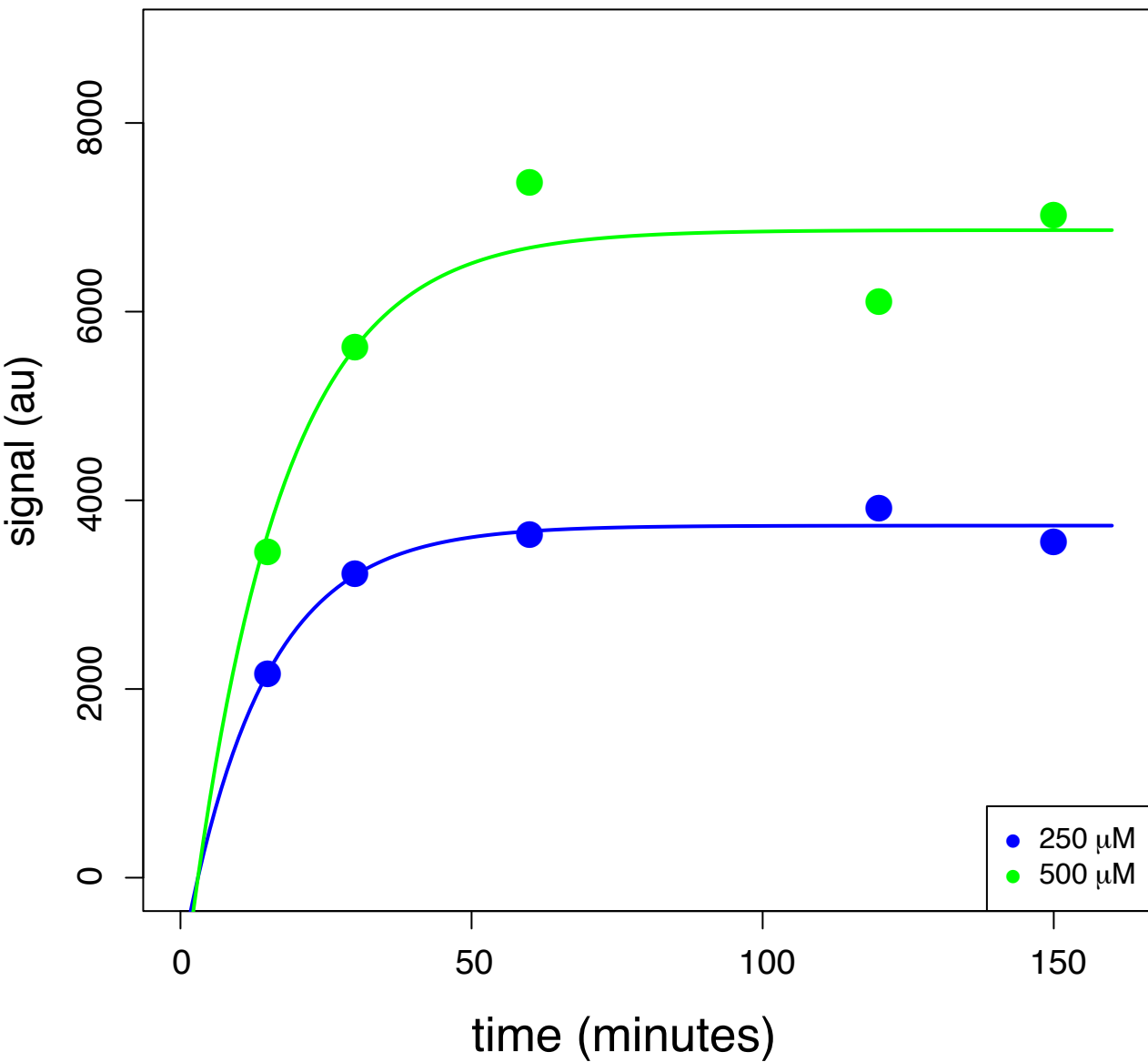

Supplement: Supplemental Material [file supp_045104.114_Figure_S6.pdf]

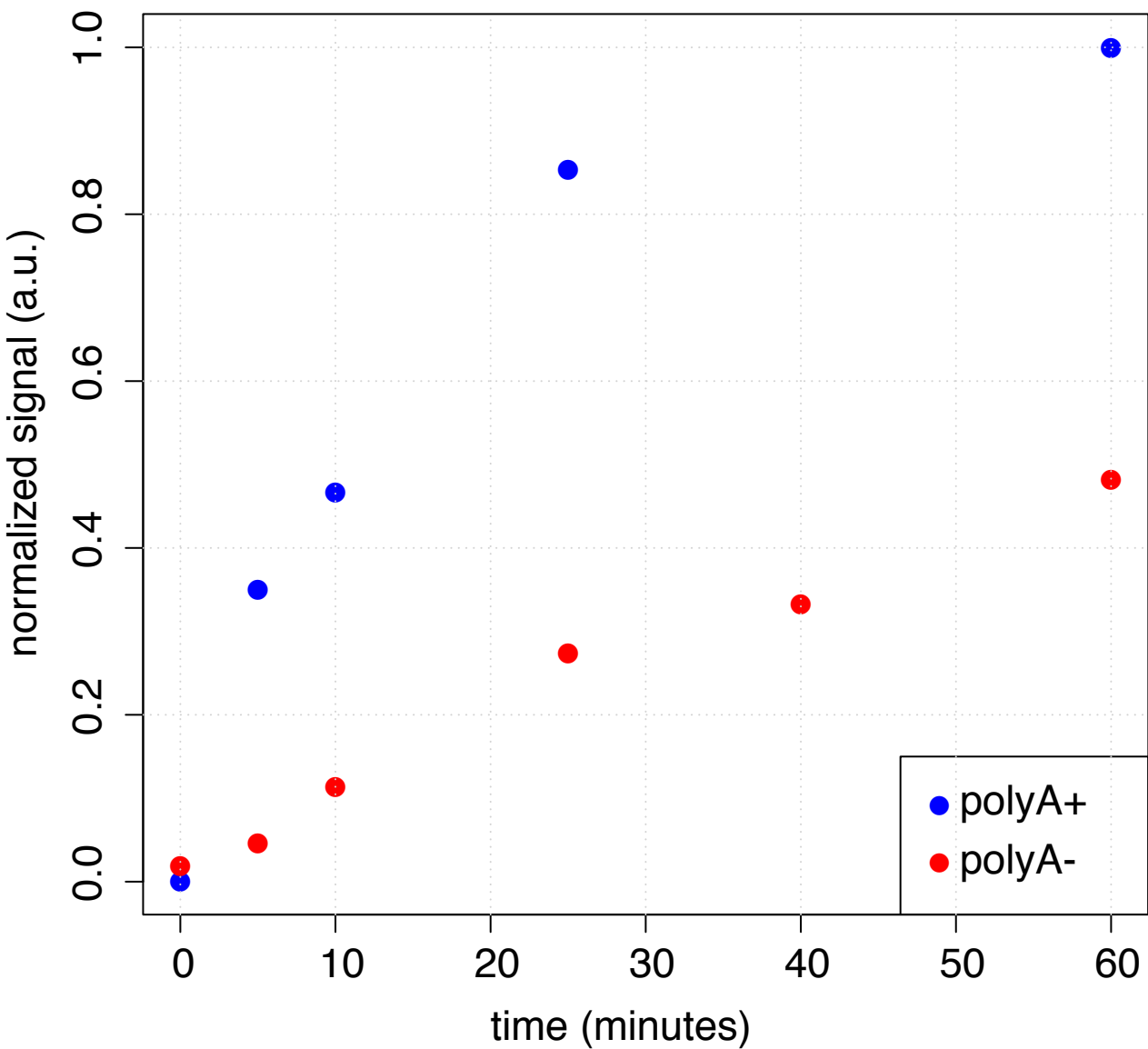

Supplement: Supplemental Material [file supp_045104.114_Figure_S7.pdf]

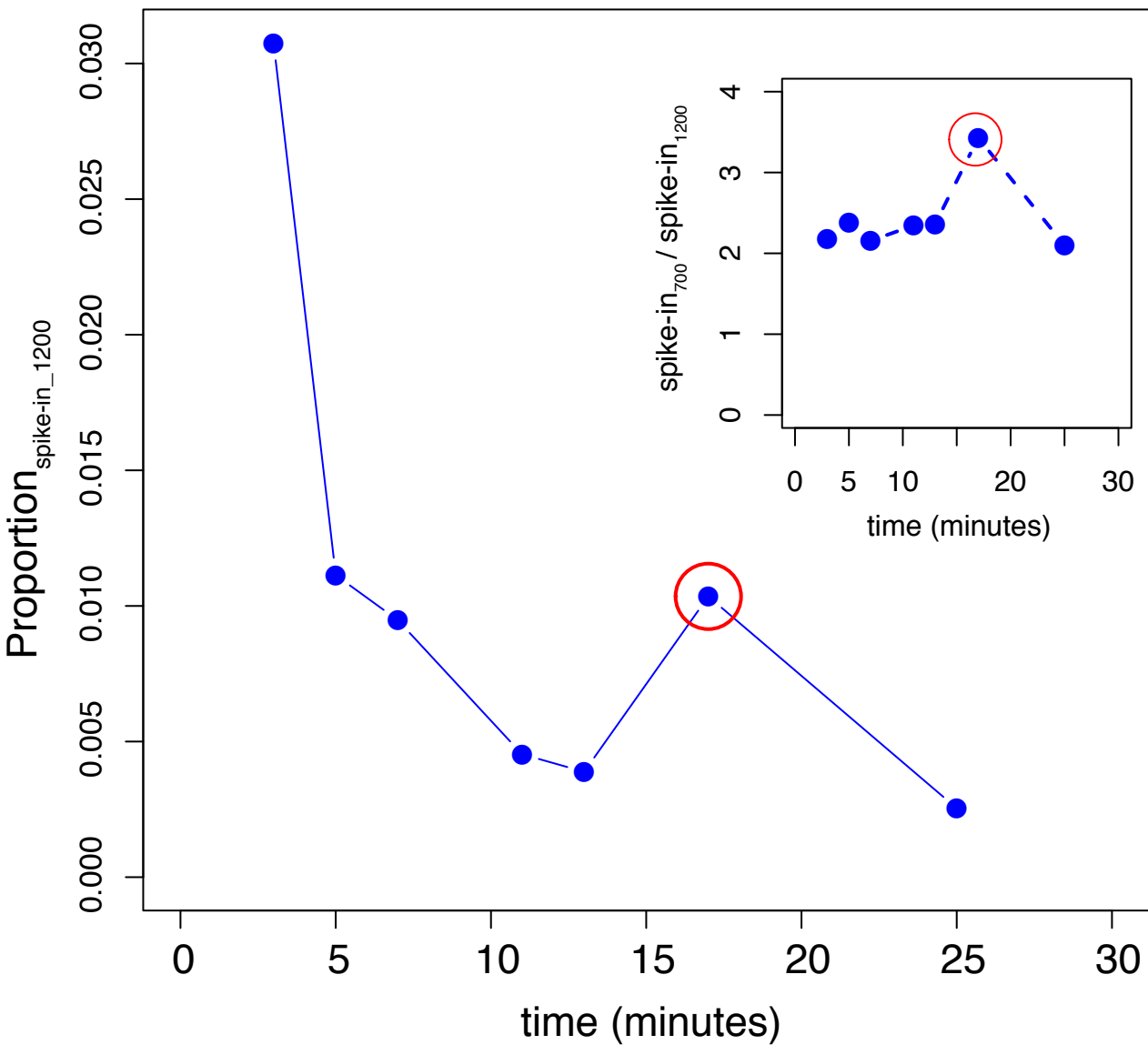

Supplement: Supplemental Material [file supp_045104.114_Figure_S8.pdf]

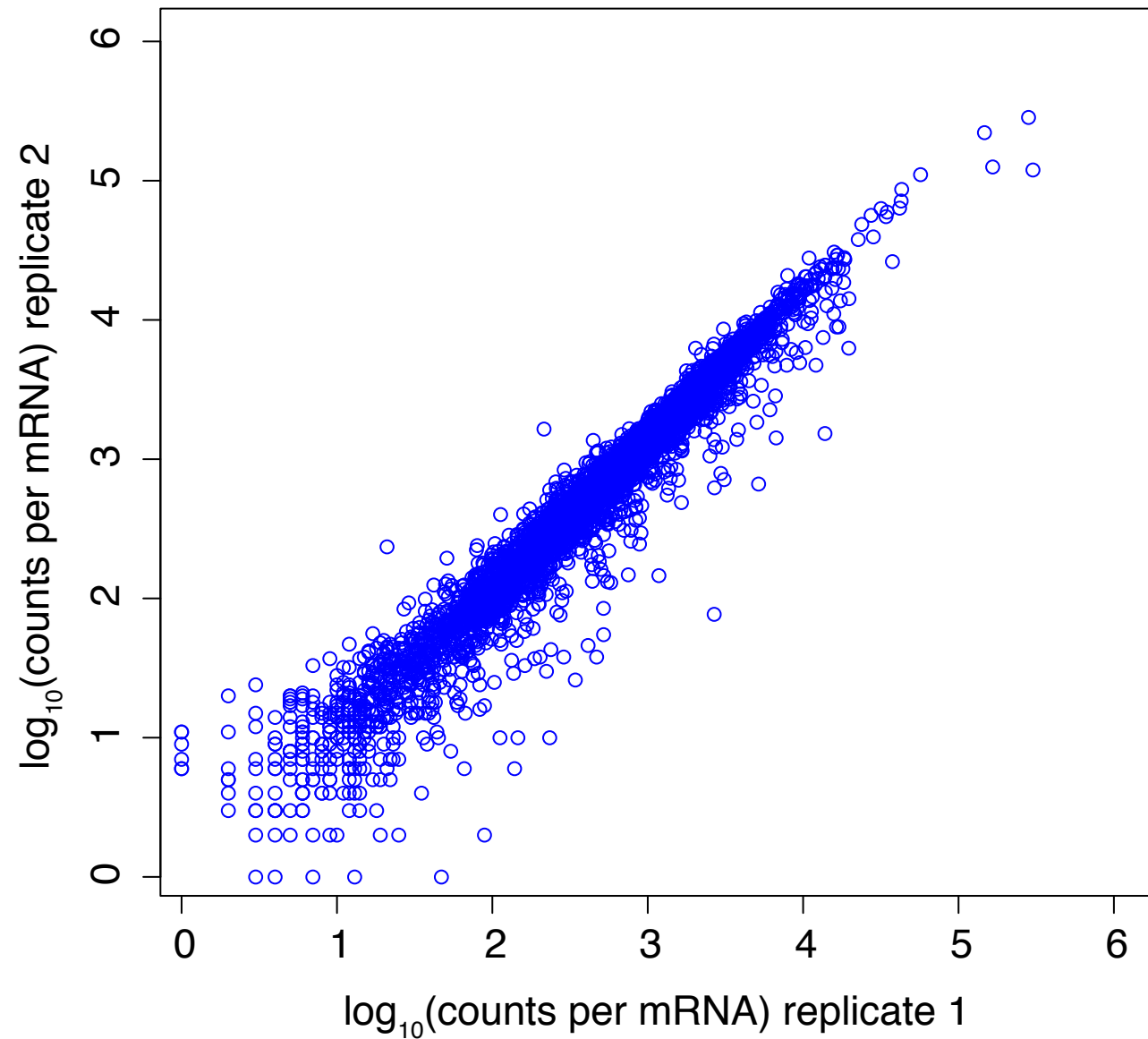

Supplement: Supplemental Material [file supp_045104.114_Figure_S9.pdf]
