# Supplementary material for: Determination of in vivo RNA kinetics using RATE-seq
Source: RNA. 2014 Oct;20(10):1645–52. doi: 10.1261/rna.045104.114 (PMC4174445; doi:10.1261/rna.045104.114)
Supplement: Supplemental Material [file supp_045104.114_TableS1.pdf]

Table S1. Sequences of three spike-in RNAs used for normalization.

>spike-in$_{700}$ 0.41 GC content
gaatacaagcttgggctgcaggtcgaccaaaaacacctgaaaaaatgttcgagcagctgggcattactgatgaatcttta
aaagcttgggacagcattacagctttcggccagctgaaagatacaaaagtacaaaaaggcgagccattgttccctcgttt
agaggcagaagaagaaattgcttacatcaaagggaaaatgcaaggttcagcaccagcgaaagaagaaacaaaagaagaag
agcctcaagaggtcgatcgtttacctgaaattacgattgatcaatttatggatgtagagcttcgcgtagctgaggtcatt
gaggcagagccagtgaaaaaagcagaccgtttattgaagctgcagcttgatcttggttttgaaaagcgccaagtggtatc
cggcattgcgaagcattatacgcctgaagagcttgttggaaaaaaactcgtttgtgtaacaaatctaaaaccggttaaac
tcagaggagagctttctcaaggtatgatccttgcaggagaagcagacggcgttttaaaggtcgtatctatcgatcagtcg
ttaccaaaaggcacaagaattaaataatgataaacaaaaggtgtttcacgtgtaacaattcgtcgaacaccttttgtgtt
tcgacaagaaaggagtttttcacttatgttgtttgacactcacgcgcatttaaatgcagaacaatatgatactgatcttg
aagaggtGGATCCCCGGGCGAGCTCCCAAAAAAAAAAAAAAAAAAAAAAAAAAAAAACCG

>spike-in$_{900}$ 0.42 GC content
gaatacaagcttgggctgcagcatcagtacgatcattcctaagctatatcctcgatttactgaaatcatacaattaattg
gagctagcggaatggtgacactgcctaccgaaaatgcatttgactcagagatacgggaagatcttgaccaatatcttcaa
gcaaccaatacaaatgctgaagaacgtgtgaaaattttccgtttggcatgggatttaacaatgagttcattcggaactag
acaaactcactacgaaagatatttttttcggagatcctattcggatttcaagcaggctgtataccagttatccaaagcagg
agcagttgaacatgattaaaacatttttacatgcagatacagaacattgaaaaaacagcgggaaaagatccgctgtttcg
tatttttattcaggaaactgaacatggcccggtactgtataggctttggacgttccgctttcaggcagctttggaatgg
tgtctttcacaacttttccgcggatgtcagtcattctgactttgagagagccagtacctaaattcgtactcacaaaatgg
ttatagtccatttttctccatgttgatccacttaccatccttttcatattccattttcataacaggatacttgtgatttct
gacttggattgctgcccaccacctgctgctgccttctttgatccggtacgtgaaattgccggtgattggggctttgacaa
cacgccatttaatattgatttttccgtctttcatattgccgattttacggaaggcattaggtgacagatcaagagctccc
cgagcgccttcgggataaagatcagtaacatatacggttgttttcccttttggcccttcaacttccaaataagagccggc
aagtgccgcttttactcctccgtaattgagatccgccggatGGATCCCCGGGCGAGCTCCCAAAAAAAAAAAAAAAAAAA
AAAAAAAAAAACCG

>spike-in$_{1200}$ 0.415 GC content
gaatacaagcttgggctgcaggcggaatcatgcactgctttacaggaagtgcggaagtggcaagagaatgcatgaaaatg
aatttttatttatcatttgggggaccggtgacatttaaaaatgcgaagaaaccgaaggaagtagtgaaggaaattcccaa
tgaccggctgctgattgaaacggactgcccgtttctcacaccgcaccctttccgcgggaaaagaaatgagccaagctatg
tgaaatatgtggcagaacaaatcgccgaattaaaagaaatgacctttgaagagattgcttccatcacgactgaaaatgcg
aaaagacttttccgtataaactgacaaaaaacgctagcgggttttgtaagagcttgtcccttacagcgtttttctataaa
agttctacatgctttcttctcctcataggataggtttgtcgacaagtctttcttccgtttctcagtgtatttcaggataa
tgaagaggacactgagcttttgggagaaagagaagggagggttgacagccttttagatactctatataatctctccgagg
agaaggaggcgtttttcatcacacaaaaaatgaaaaagctgttttccgtaaagcttagcaaaagcaaagtcattctggtt
gctgcttgtttgcttttggcaggaagcgggactgcgtacgcggctcatgagctgacgaaacaatcagtctcagtttctat
caatggcaaaaagaaacatatacgcacacatgcaaatacagtcggtgatcttttggagacgcttgatataaagacaagag
acgaagacaagatcacacctgctaaacagacaaagataacagcagacatggacgttgtgtatgaggctgcaaaacctgtg
aagcttacaataaacggggaagaaaagacgttatggtcaacagcaaaaacggtcggtgcattactggacgaacaagatgt
tgatgtgaaagaacaagatcaaattgatcccgcaatagatacagatatttcgaaagacatgaagattaacatagagcccg
catttcaggttactgtgaatgatgcagggaaacaaaagaagatctggacgacttcgactacggtcgctgactttttaaaa
cagcaaaagatgaacataaaagacgaagataaaatcaagcctgcgttagatgcaaagctgacgaaaggaaaggctgatat
tacaattactcgtatcgaaaaGGATCCCCGGGCGAGCTCCCAAAAAAAAAAAAAAAAAAAAAAAAAAAAAACCG
