# Supplementary material for: Determination of in vivo RNA kinetics using RATE-seq
Source: RNA. 2014 Oct;20(10):1645–52. doi: 10.1261/rna.045104.114 (PMC4174445; doi:10.1261/rna.045104.114)
Supplement: Supplemental Material [file supp_045104.114_SuppLegends.docx]

**SUPPLEMENTARY INFORMATION**

**Figure S1: Thiouracil is taken up by prototrophic and auxotrophic cells with equal efficiencies.** The absorbance spectra of equivalent amounts of RNA purified from a *ura3^-^* and prototrophic strain grown in the presence of uracil and either the presence or absence of 4sU. 4sU has maximal absorption at 327nm resulting in a unique peak of absorbance in RNA labeled with 4sU (top inset). 2µg of the same total RNA sample from an auxotrophic cell grown in the (i) absence and (ii) presence of 4sU was compared to the same amount of RNA isolated from a prototrophic strain grown in the (iii) absence and (iv) presence of 4sU.

**Figure S2. Thiouracil cannot supplement a uridine monophosphate auxotroph.** Cell densities were determined following 24 hours growth in the presence of 100µM uracil and different concentrations of 4sU. The presence of 4sU has no visible effect on final culture density for a prototrophic strain (blue diamonds) or an auxotrophic strain (brown circles). A *ura3^-^* strain does not grow when supplemented with 4sU in the absence of uracil (orange circle).

**Figure S3: Thiouracil has no detectable effect on cell growth for the duration of labeling.** Cells in log phase growth supplemented with 500µM uracil were treated with 500µM 4sU (blue curve) or an equivalent volume of DMSO (red curve). Error bars correspond to 2 standard errors of the mean (95% confidence interval) of two biological replicates and at least two technical measurements of each.

**Figure S4: Thiouracil minimaly affects cell growth at higher concentrations and longer durations of labeling**. Cells were grown in 500µM uracil and varying concentrations of 4sU. Measurement of changes in cell density with time was initiated after at least two population doublings. The presence of 4sU seems to have a slight effect on rate of growth with increasing exposure times and higher concentrations.

**Figure S5: Effect of 4sU exposure on steady-state transcript levels.** RNA from cells treated with 4sU for an extended period of time was compared to RNA from cells never exposed to 4sU. Microarray analysis shows that in both (**a**) a strain defective in UMP biosynthesis (*ura3^-^*) and (**b**) a prototrophic strain, the differences in steady state RNA levels are minimal, with 99% of transcripts having a log_2_ fold change from-1 to 1 (**Table S7**).

**Figure S6: The extent of 4sU labeling is concentration and time dependent.** The kinetics of the approach to equilibrium is essentially the same for total RNA grown in the presence of 250µM or 500µM 4sU. The expected difference in the final steady state level of labeled RNA is the only observed difference.

**Figure S7: Total RNA and mRNA have distinct labeling kinetics.** Equivalent masses of biotinylated polyadenylated RNA and non-polyadenylated RNA were bound to a nitrocellulose membrane using a dot blot and visualized with streptavidin alkaline phosphatase and chemifluorescence. The signal for each RNA sample was normalized to the maximal signal following 24 hours of growth. Non-polyadenylated RNA, which is primarily rRNA, approaches its equilibrium value more slowly than polyadenylated mRNA.

**Figure S8: Identification of technical biases in library preparation using multiple spike-ins**. In our experiments, we expect the relative abundance of each spike-in to decrease with time. We also expect that the ratio between spike-ins, which have been added from the same pool, should be relatively constant across time. We found that at timepoint t=17 minutes spike-in_1200_ is deviant from its expected behavior (shown) as are spike-in_700_ and spike-in_900_ (not shown). Simultaneously, the ratio between spike-in_700_ and spike-in_1200_ (inset) is relatively uniform across time, with the exception of t=17 minutes. These two metrics suggest that technical biases in library preparation have been introduced in this sample, and the timepoint was excuded from further analysis.

**Figure S9. Total counts per transcript are highly correlated between replicates.** Total counts per mRNA were compared for replicate experiments at the same time point (5 minutes) following label addition. The Spearman correlation between replicates is ρ=0.98.

**Figure S10. Normalized transcript count data are over-dispersed.**  The mean-variance relationship for normalized data. The expectation from a Poisson distribution of equality between transcript mean and variance is indicated by the red line.

**Figure S11**. **Comparison of estimated RNA half-lives between our studies and previous publications.** The Pearson correlation between our study and (**a**) (Wang et al. 2002), (**b**) (Grigull et al. 2004), (**c**) (Shalem et al. 2008), (**d**) (C. Miller et al. 2011), and (**e**) (Munchel et al. 2011) is indicated. The Pearson correlation between (Wang et al. 2002) and (**f**) (Grigull et al. 2004), (**g**) (Shalem et al. 2008), (**h**) (C. Miller et al. 2011), and (**i**) (Munchel et al. 2011) is indicated.

**Figure S12: The effect of nucleotide recycling on pulse-chase labeling**. The population of labeled transcripts can be modeled by the equation *dY/dt = xβ -αY* where *α* is the degradation rate constant, *Y* is the number of transcripts, *β* is the rate of synthesis, and *x* is a coefficient ranging from 0-1 denoting the fraction of newly synthesized transcripts that are labeled. If x=1 all new transcripts are labeled following the chase whereas when x=0 no newly synthesized transcripts incorporate the label following the chase. In the case of a pulse-chase experiment one starts with a labeled population of transcripts. In the scenario of a perfect chase, x=0, and the observed rate of mRNA degradation (red) is the same as that estimated using approach to equilibrium (blue curve), which does not depend on synthesis kinetics. However, if the label is recycled then x>0 and the label will be incorporated into newly synthesized transcripts following the chase. For example, if x = 0.1, 0.2, or 0.3 the degradation rate is under-estimated by 29% (brown curve), 45% (green curve), and 57% (purple curve) respectively.

**Figure S13:** **Genome-wide degradation rate estimates for the ribosomal protein (RP) regulon are most similar based on method**. We compared methods using transcriptional shut-off (Wang et al. 2002; Grigull et al. 2004; Shalem et al. 2008) with methods using 4sU labeling (Munchel et al. 2011; C. Miller et al. 2011) including the current study. The study is indicated by the first author's name. (**a**) Using transcriptional shut off, transcripts belonging to RP regulon (pink) are found to have half-lives similar to the entire transcriptome (blue) whereas (**b**) all estimates using *in vivo* metabolic labeling find that the RP transcripts are long-lived relative to the rest of the transcriptome.

**Figure S14. Comparison of mRNA synthesis rates estimates using RATE-seq and GRO.**

We compared rates of synthesis using RATE-seq with those estimated using Genomic Run On (Pelechano, Chávez, and Pérez-Ortín 2010). The estimates are positively correlated with Spearman correlation coefficient ρ=0.50.

**Figure S15: Optimization of 4sU labeling protocol**. Each step of the 4sU labeling procedure was optimized as follows. (**a**) We determined that the quantity of biotin-HPDP used for conjugating to 4sU-labeled RNA was sufficient by using a fixed amount of biotin-HPDP with differing quantities of 4sU-labeled RNA. There is no decrease in signal with increasing quantities of RNA using a colorimetric dot blot analysis indicating that biotin-HPDP is in excess for the range of RNA concentrations used in RATE-seq. (**b**) We optimized the quantity of streptavidin-labeled beads to ensure that the entire labeled fraction is captured. RNA that was not captured by the streptavidin-labeled beads was assayed for 4-sU content using a colorimetric dot blot assay. We determined that 200µl of beads is sufficient to capture all labeled RNA. (**c**) We determined the pull down efficiency of samples treated with 4sU as compared with untreated cells by quantifying the total amount of recovered RNA following biotin-HPDP conjugation and streptavidin beads of a fixed mass of RNA. For an equal quantity of RNA, the yield of RNA is 100-fold higher for the labeled samples compared with unlabeled sample in both a *ura3^-^* auxotroph and a prototrophic strain.

**Figure S16: Labeling bias in 4sU treatment**. Comparison of coding sequence length with the relative microarray signal (log_2_) (**Table S7**) from fractionated thiolated RNA compared to the unfractionated sample indicates that longer transcripts are preferentially labeled and/or recovered.

**Figure S17: Sequencing read counts are positively correlated with the amount of uracil in a transcript**. The total number of uracils in a transcript is a function of its length. A longer transcript typically has more counts at each time point during the labeling as a result of both increased label incorporation and increased transcript length. The relationship between uracil number in a transcript and RNA-seq counts is apparent at (**a**) 5, (**b**) 13, (**c**) 25, and (**d**) 100 minutes after labeling.

**Figure S18: Labeling bias affects the equilibrium value for each gene.** The steady state equilibrium value (Y_eq_) estimated by our non-linear modeling is positively correlated with the total number of sequence counts per transcript at each time point. The relationship between RNA-seq counts and Y_eq_ is apparent at (**a**) 5, (**b**) 13, (**c**) 25, and (**d**) 100 minutes after labeling.

**Figure S19: Labeling bias does not influence the estimated degradation rate constant.** The estimated degradation rate constant (α) is not correlated with the number of counts for each transcript at any time point during the approach to equilibrium. There is no relationship between RNA-seq counts and α detectable after (**a**) 5, (**b**) 13, (**c**) 25, and (**d**) 100 minutes of labeling.

**Table S1. Sequences of three spike-in RNAs used for normalization.**

**Table S2. Unprocessed read counts for replicate 1.**

**Table S3. Unprocessed read counts for replicate 2.**

**Table S4. Normalized read counts used for model fitting.**

**Table S5. Kinetic parameters of RNA synthesis and degradation estimated using RATE-seq.**

**Table S6. Functionally related transcripts have similar decay rates.**

**Table S7. Microarray data assessing labeling bias.**
