# Supplementary material for: Determination of in vivo RNA kinetics using RATE-seq
Source: RNA. 2014 Oct;20(10):1645–52. doi: 10.1261/rna.045104.114 (PMC4174445; doi:10.1261/rna.045104.114)
Supplement: Supplemental Material [file supp_045104.114_rateSeqFit.R]

rateSeqFit 

# Determination of in vivo RNA kinetics using RATE-seq
